# Effect of Chronic Escitalopram versus Placebo on Personality Traits in Healthy First-Degree Relatives of Patients with Depression: A Randomized Trial

**DOI:** 10.1371/journal.pone.0031980

**Published:** 2012-02-29

**Authors:** Ulla Knorr, Maj Vinberg, Erik Lykke Mortensen, Per Winkel, Christian Gluud, Jørn Wetterslev, Ulrik Gether, Lars Vedel Kessing

**Affiliations:** 1 Psychiatric Centre Copenhagen, Rigshospitalet, University Hospital of Copenhagen, Copenhagen, Denmark; 2 Copenhagen Trial Unit, Centre for Clinical Intervention Research, Rigshospitalet, University Hospital Copenhagen, Copenhagen, Denmark; 3 Department of Health Psychology, University of Copenhagen, Copenhagen, Denmark; 4 Department of Neuroscience and Pharmacology, Centre for Pharmacogenomics, University of Copenhagen, Copenhagen, Denmark; University of Wuerzburg, Germany

## Abstract

**Introduction:**

The serotonergic neurotransmitter system is closely linked to depression and personality traits. It is not known if selective serotonin reuptake inhibitors (SSRI) have an effect on neuroticism that is independent of their effect on depression. Healthy individuals with a genetic liability for depression represent a group of particular interest when investigating if intervention with SSRIs affects personality. The present trial is the first to test the hypothesis that escitalopram may reduce neuroticism in healthy first-degree relatives of patients with major depressive disorder (MD).

**Methods:**

The trial used a randomized, blinded, placebo-controlled parallel-group design. We examined the effect of four weeks escitalopram 10 mg daily versus matching placebo on personality in 80 people who had a biological parent or sibling with a history of MD. The outcome measure on personality traits was change in self-reported neuroticism scores on the Revised Neuroticism-Extroversion-Openness-Personality Inventory (NEO-PI-R) and the Eysenck Personality Inventory (EPQ) from entry until end of four weeks of intervention.

**Results:**

When compared with placebo, escitalopram did not significantly affect self-reported NEO-PI-R and EPQ neuroticism and extroversion, EPQ psychoticism, NEO-PI-R openness, or NEO-PI-R conscientiousness (p all above 0.05). However, escitalopram increased NEO-PI-R agreeableness scores significantly compared with placebo (mean; SD) (2.38; 8.09) versus (−1.32; 7.94), p = 0.046), but not following correction for multiplicity. A trend was shown for increased conscientiousness (p = 0.07). There was no significant effect on subclinical depressive symptoms (p = 0.6).

**Conclusion:**

In healthy first-degree relatives of patients with MD, there is no effect of escitalopram on neuroticism, but it is possible that escitalopram may increase the personality traits of agreeableness and conscientiousness.

**Trial Registration:**

Clinicaltrials.gov NCT00386841

## Introduction

Neuroticism seems to reflect an enduring vulnerability to major depressive disorder (MD) partly reflecting shared genetic risk factors [1]. Most of the genetic risk for MD expressed via personality is captured by neuroticism, with a modest influence of conscientiousness, and with small influence of openness, extroversion, and agreeableness [2], [3]. When neuroticism decreases in patients with depression treated with antidepressants, it has been difficult to clearly distinguish the treatment effect on neuroticism from the treatment effect on the depressive disorder, as remission of depressive symptoms is associated with partial normalization of neuroticism [4]. Decrease in neuroticism scores during paroxetine treatment of patients with MD, even after controlling for depression improvement, has been observed in a large group of depressed patients [4]. Thus, it is possible that response to selective serotonin re-uptake inhibitors (SSRIs) may be mediated at least partly via a decrease in neuroticism [4], [5]. Higher neuroticism is associated with higher thalamic serotonin binding [6]. Furthermore, a recent study from our group has suggested that familial risk of depression and neuroticism interact in their relation to the degree of specific serotonin transporter binding [7]

Two randomized trials have investigated the effect of SSRI on behavior and aspects of personality with some relation to neuroticism in healthy participants without a family history of MD. Knutson et al. found that four weeks intervention with paroxetine 20 mg/day (n = 26) versus placebo (n = 25) significantly increased social affiliation and decreased negative affect [8]. Tse et al. found that two weeks intervention with citalopram 20 mg/day (n = 11) compared with placebo (n = 9) resulted in a statistically significant increase in self-directedness [9]. Results from these trials suggest that SSRIs may affect aspects of personality even in the absence of clinical depression. SSRIs do not seem to influence mood in healthy individuals [10]–[13]. Results from a number of studies, although not all [14], have suggested increased levels of neuroticism in healthy first-degree relatives of patients with MD compared to healthy individuals without a family history of MD [15]. However, no trial has investigated the effect of SSRIs on neuroticism and other personality dimensions in healthy individuals with a family history of MD [16].

Thus, to examine the effect of escitalopram on neuroticism and other aspects of personality, and excluding an effect on depression, we recruited healthy first-degree relatives of patients with MD for the AGENDA (**A**ssociations between **G**ene-polymorphisms, **E**ndophenotypes for **D**epression and **A**ntidepressive Intervention) trial. The trial is the first to investigate the effect of a four week self-administered daily escitalopram versus placebo on personality traits, as measured with the Revised Neuroticism-Extroversion-Openness-Personality Inventory (NEO-PI-R) and the Eysenck Personality Inventory (EPQ), in healthy individuals . We tested the hypothesis that intervention with escitalopram for a month compared with placebo decreases symptoms of neuroticsm in healthy first-degree relatives of patients with MD.

## Materials and Methods

### Study design and Ethics Statement

The AGENDA trial was investigator initiated and designed. The protocol for this trial and supporting CONSORT checklist are available as supporting information; see Checklist S1 and Protocol S1. It was conducted as a participant-, investigator-, observer-, and data-analyst-blinded trial. During the trial the participants received either escitalopram 10 mg/day or placebo for a period of four weeks. The trial was conducted from July 2007 until July 2009 at the Department of Psychiatry, Rigshospitalet (now Psychiatric Centre Copenhagen), Denmark as part of the Centre for Pharmacogenomics, University of Copenhagen, Denmark. The trial was conducted and monitored in accordance with the International Conference on Harmonization for Good Clinical Practice guidelines and principles expressed in the Declaration of Helsinki. The Local Ethics Committee (De Videnskabsetiske Komitéer for Københavns og Frederiksberg Kommuner, Københavns Kommune) approved the trial: H-KF 307413.

An independent data monitoring and safety committee was established to further ensure the safety of the participants, should the need have occurred for early stopping. All participants gave written informed consent. The detailed trial protocol was published ahead of study completion and the changes in neuroticism scale scores on the NEO-PI-R and the EPQ were pre-defined outcomes [17]. Results on the primary outcome change in the area under the curve for cortisol measurements during the combined dexamethasone-corticotropin releasing hormone test have been reported elsewhere [18], thus neuroticism was a secondary outcome in addition to cognition published in [19].

### Probands and participants

**Figure 1:**
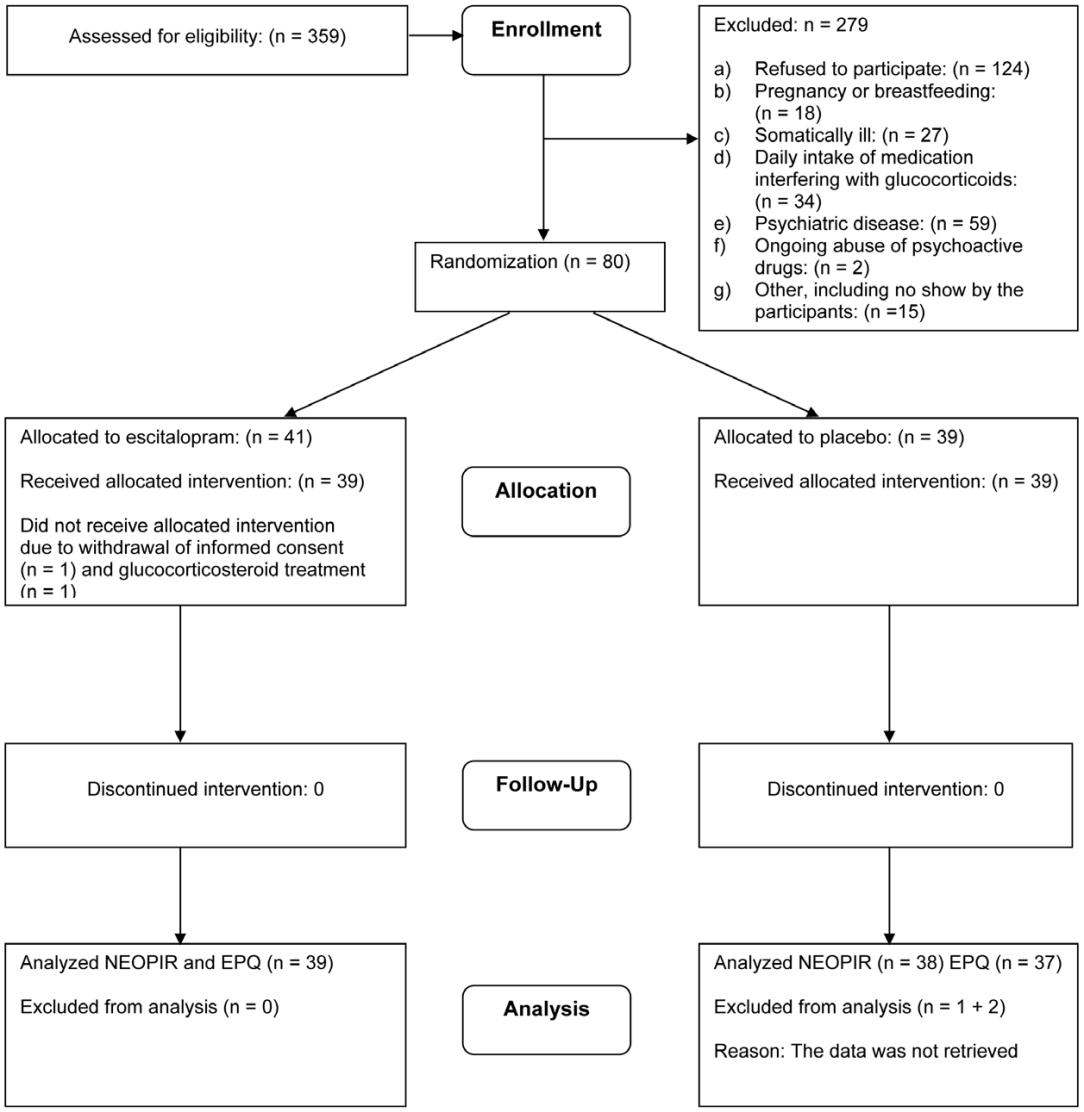
Flowchart consort for Agenda Trial Neuroticism.

The selection of diseased probands and healthy participants has previously been described in details [17]. Probands were patients with a diagnosis of MD from psychiatric hospital in- or out-patient contact in Denmark who participated in ongoing studies at the Department of Psychiatry, Rigshospitalet, Denmark. The proband's diagnoses were validated by face-to-face interviews including the semi-structured interview Schedules for Clinical Assessment in Neuropsychiatry (SCAN) by trained clinicians [20]. Probands were asked to permit a contact to their adult children and/or siblings who were the eligible participants for the AGENDA trial. The probands (n = 466) gave written permission to contact 359 first-degree relatives, who were the potential participants in the trial. The participant flow is shown in Figure 1.

Individuals of either sex, aged 18–60 years with Danish ethnicity (i.e., born in Denmark with Danish parents and European grandparents) were eligible for the trial. Ethnicity was used to get a genetically homogeneous sample. We excluded individuals with somatic illnesses or a handicap that made participation in the trial impossible while six individuals with stable, treated milder medical conditions were included: hypertensio arterialis (three), pancreatitis antea (one), hypothyroidism (one), and acne vulgaris (one). Furthermore, we excluded individuals with a daily intake of drugs interfering with corticosteroids or escitalopram (cipralex), including birth control pills or any kind of corticosteroids, and individuals who were allergic to the study drug or placebo. Additionally, former medical or psychological treatment for diseases in the affective or schizophrenic spectrum and current abuse of alcohol or psychotropic medication led to exclusion. Women who were trying to conceive, or who were pregnant or breastfeeding were excluded. Women were postmenopausal or in the lutheal phase of the menstrual cycle at the time of randomization. Women taking birth control pills were instructed to discontinue these six weeks prior to entering the trial. Furthermore, all women were carefully instructed to use double barrier birth control methods and pregnancy tests were performed both before and after the intervention [17].

### Trial procedures

Escitalopram and placebo tablets were identical in appearance, color, smell, taste and solubility allowing for blinding of the assignment to intervention or placebo. H. Lundbeck A/S provided identically appearing blister packages containing escitalopram or placebo. An independent pharmacist then packed, sealed, and numbered the drug packages according to a randomization list provided and concealed by the Copenhagen Trial Unit (CTU). The participants were randomized to self-administer a single dose of either escitalopram 10 mg or placebo each evening for four weeks. On completion of four weeks of double-blind intervention participants entered a five-day blinded down-titration period to nil medication. Adherence to the protocol was sought by making weekly telephone calls to the enrolled participants. Following four weeks of intervention the participants were asked, how adherent they had been to the protocol, and if they had missed taking any tablets.

The sample size was estimated according to the primary outcome as previously described [17]. The CTU conducted the centralized computerized randomization by telephone to secure adequate allocation sequence generation and allocation concealment [21]. Randomization was stratified in blocks by age (18–31 years and 32–60 years) and sex. Only the data manager knew the block size, which was six. Participants were randomized 1∶1 to receive either escitalopram 10 mg or placebo. Randomization was done immediately after a face-to-face interview including the SCAN-interview at the first scheduled appointment at the clinic establishing that a participant fulfilled all the inclusion criteria and none of the exclusion criteria. All trial personnel and participants were blinded to the packaging of the study drug, and blinding was maintained throughout monitoring, follow-up, data management, assessment of outcomes, data analyses, and drawing of conclusions. Then the randomization code was broken. The blinding was successful as previously described [18].

### Assessments

The first part of the assessment was a telephone interview, and eligible individuals were scheduled to meet at the clinic on two different days both before and following the four weeks of intervention. On the first day of examination the participants gave written informed consent after details of the trial were explained. We did a SCAN interview (UK) and individuals with a prior or current psychiatric diagnosis were excluded (n = 59, see Figure 1). Further assessment included information on family history of psychiatric disorders, ratings of mood using the 17-item Hamilton Depression Rating Scale (HAM-D) [22], and the 14-item Hamilton Anxiety Scale [22], self-rated depressive symptoms by Beck Depression Inventory, 42-items [23], various socio-demographics, height, weight, routine blood tests, and, a pregnancy test for women. Furthermore, blood was drawn for measurements of plasma escitalopram, and the UKU Side Effect Rating Scale [24], was applied by the principal investigator after four weeks of intervention.

### Assessment of neuroticism

The personality dimension neuroticism was assessed by the Danish version of Eysenck Personality Questionnaire (EPQ) [25], [26], and the Revised Neuroticism-Extroversion-Openness-Personality Inventory (NEO-PI-R) [27]. The EPQ comprises 101 yes-no items that measure the broad dimensions of neuroticism, extroversion, and psychoticism. NEO-PI-R is a 240-items inventory that evaluates the broad personality dimension of neuroticism, extraversion, openness, agreeableness, and conscientiousness. The score on each of the five broad dimensions was derived by adding the scores from the assessments of six constituent personality traits (facets). The respondent answers the statements on a 5-point Likert scale from ‘disagree very much’ to ‘agree very much’. Both EPQ and NEO-PI-R were applied before (entry) and following four weeks of intervention (4 weeks).

### Measure of plasma escitalopram

Escitalopram was extracted and quantitated was carried out on an ASPEC XL combined with a HPLC system, both from Gilson, Villiers le Bell, France. Lower and upper limits of quantitation were 10 and 3,600 nmol/l, respectively. Imprecision ranged from 5.5% to 8.4% and trueness ranged from 93.2% to 103.0% within the measurement range. Extraction recovery was 38% and carry-over was less than 1%.

### Statistical methods

The data analyses planned for the secondary outcomes were described in a pre-established statistical analysis plan [17]. The null-hypotheses to be tested were that there would be no difference between the two intervention arms with regard to changes in neuroticism assessed by the EPQ and NEO-PI-R. The outcomes were changes in personality scores calculated as the score at week 4 minus the score at entry for the individual participants. Firstly, independent samples t-tests were used to compare change scores in the escitalopram and placebo groups for NEO-PI-R neuroticism and EPQ neuroticism. Secondly, adjustments were planned to be conducted for age, sex, years of education, and concentration of escitalopram in plasma in a general linear model if these variables were associated with change in neuroticism at the 0.1 level of significance [17].

## Results

A total of 80 participants were randomized. The characteristics of the participants can be seen in Table 1. The mean age of non-participants was 37 (SD11) years and 58% were women. The reasons for non-participation are presented in Figure 1. A statistically significant correlation was found between EPQ neuroticism and NEO-PI-R neuroticism reported at entry (Pearsson = 0.8; p<0.0005). There was no statistical significant difference in the change of HAM-D (four weeks - entry) between the escitalopram group and the placebo group (p = 0.6). No severe adverse reactions or serious adverse events were obseved. The side effect measure UKU total for participants of the escitalopram and placebo groups did not differ significantly at four weeks (Table 1). However, sexual adverse effects showed a statistically significant increase and insomnia showed a statistically significant decrease in the escitalopram group compared with the placebo group [18].

**Table 1:** Clinical and demographic characteristics of participants in the Agenda Trial.

Characteristic	Escitalopram(n = 41)	Placebo(n = 39)	All Participants(n = 80)
Age – yr, mean ± SD	32±11	31±11	32±10
Women – N (%)	15 (37)	14 (36)	29 (36)
Education – mean ± SD			
Years of school	11±1	11±1	11±1
Years of further education	3±2	3±2	3±2
First degree relatives of patient with a history of major depressive disorder– median (25,75 quartiles)	1 (1;2)	1 (1;2)	1 (1;2)
17-item Hamilton Depression Scale Score – median (quartiles) (range)	1 (0;3)(0–7)	1 (0;3)(0–7)	1 (0;3)(0–7)
Beck Depression Inventory, 21-item, depression – median (25,75 quartiles)	2 (0;4)	2 (0;3)	2 (0;5)
Side Effects UKU total score (4 weeks), median (25,75 quartiles), (range)	1(0–4), (0–13)	0(0–2), (0–7)	0(0–3), (0–13)
Plasma escitalopram (4 weeks), nmol/l,	50±29		
- mean ± SD, median (range)	48 (<10–138)	<10	
Eysenck (entry)- mean ± SD, median (25,75 quartiles)			
	6.8±5.3	7.3±4.4	7.0±4.8
Neuroticism	7 (1.5;10)	6.0 (4;10)	6.5 (3;10)
	16.0±3.8	14.7±4.5	15.4±4.2
Extraversion	17 (14.5; 18.5)	17 (12;18)	17 (14;18)
NEO-PI-R (entry)- mean ± SD, median (25,75 quartiles)			
	68±24	71±18	70±21
Neuroticism	66 (50;85)	70 (59–85)	68 (55;85)
	125±19	123±16	124±18
Extraversion	123 (110;138)	125 (111;136)	123 (110;136)
	114±17	118±18	116±74
Openness	114 (99;125)	120 (106;131)	115 (100;130)
	124±18	128±12	126±54
Agreeableness	125 (118;136)	128 (119;138)	127 (118;137)
	114±20	113±17	114±18
Conscientiousness	115 (104;133)	111 (102;124)	112 (102;126)

### Effects on neuroticism

The dataset was complete with the exception of one man and one woman in the escitalopram group, who left the trial prior to the intervention, and two men in the placebo group in whom data collection failed for both EPQ and NEO-PI-R in one and for EPQ in another, see Figure 1. There was no statistical significant difference in the change in reported neuroticism scores according to the NEO-PI-R (p = 0.09) and the EPQ (p = 0.7) for participants who got escitalopram compared to those who got placebo (Table 2). No stastistically significant correlations were found between change in neuroticism measured using EPQ or NEO-PI-R, and age, sex, years of education, or plasma escitalopram. The exclusion of two participants with immeasurable concentrations did not change the results substantially.

**Table 2:** Changes in personality scores in the escitalopram and the placebo group following four weeks of treatment.

Personality trait(4 weeks -entry)	Interventiongroup	Mean (SD)	Median	Minimumvalue	Maximumvalue	Interquartile range	*p*
Neuroticism^c^	Escitalopram	−1.77 (3.74)	−1	−9	12	4	
	Placebo	−2.08 (2.86)	−2	−9	4	4	0.73^b^
Neuroticism^d^	Escitalopram	−3.01 (10.3)	−4	−31	19	10	
	Placebo	1.00 (10.5)	1	−21	27	16	0.09^a^
Extraversion^d^	Escitalopram	1.51 (7.95)	2	−16	18	10	0.90^a^
	Placebo	1.32 (6.24)	2	−15	15	8	
Openness^d^	Escitalopram	3.18 (9.84)	5	−30	20	8	0.33^b^
	Placebo	2.15 (9.97)	3	−17	38	14	
Agreeableness^d^	Escitalopram	2.38 (8.09)	1	−18	19	11	0.046^a^
	Placebo	−1.32 (7.94)	−3	−15	18	11	
Conscientiousness^d^	Escitalopram	1.85 (8.41)	2	−12	20	14	0.07^a^
	Placebo	−2.34 (11.4)	−1	−42	14	14	

a ) The distributions did not differ significantly from the normal distribution (Shapiro Wilkes test) and a t-test was used to compare the escitalopram and the placebo arm. P of Levene's test ranged from 0.11 to 0.80.

b ) The distributions differed from the normal distribution but judged from the graphical displays (histograms and probability distributions) they followed normal distributions with reasonable approximation, thus a t-test was also used.

c ) Eysenck: Escitalopram (n = 39), placebo (n = 37).

d ) NEO-PI-R: Escitalopram (n = 39), placebo (n = 38).

Further analyses showed no statistically significant correlations between: change in EPQ neuroticism and BDI-21 at entry (rho = −0.26; p = 0.06), change in EPQ neuroticism and HAM-D at entry (rho = 0.12; p = 0.3), change in NEO-PI-R neuroticism and BDI-21 at entry (rho = −0.10; p = 0.4), and change in NEO-PI-R neuroticism and HAM-D at entry (rho = −0.05; p = 0.7).

Furthermore, no statistically significant differences were shown in changes in EPQ extraversion (p = 0.2), EPQ psychoticism (p = 1.0), NEO-PI-R extraversion (p = 0.9), NEO-PI-R openness (p = 0.3), and NEO-PI-R conscientiousness (p = 0.07) between escitalopram and placebo participants. However, a statistically significant difference was found in the change in NEO-PI-R agreeableness between escitalopram (mean: 2.38, SD; 8.09) and placebo (mean −1.32, SD: 7.94; p = 0.046 (Table 2).

## Discussion

The results of the AGENDA trial do not support our hypothesis that a four-week long intervention with escitalopram as compared with placebo would decrease EPQ or NEO-PI-R neuroticism total scores in healthy first-degree relatives of patients with MD. However, we found statistically significant changes on the agreeableness dimension of personality on the NEO-PI-R, (p = 0.046) but none of the other dimensions of personality measured by EPQ or NEO-PI-R were significantly affected by the intervention. We found a trend for change in NEO-PI-R conscientiousness (p = 0.07) between escitalopram and placebo participants. We had no hypothesis regarding conscientiousness, and greater statistical power might have been needed to evaluate this item. We found no significant correlation between change in neuroticism during intervention, and age, sex, education or plasma escitalopram concentration, respectively.

Our present trial is the first to suggest an effect of escitalopram on agreeableness in healthy individuals without depressive symptoms. The trial has several advantages. The participants were studied in a randomized clinical trial blinded in all phases including the statistical analyses and conclusion phase. The trial and the analyses were carried out as planned in advance and the completion and compliance in the trial was very high. The registered diagnosis of depression for the probands was verified by a face-to-face psychiatric research interview by trained medical doctors. The participants were assessed and diagnosed by validated and frequently used multi-dimensional methods. Further, the participants were subjected to four weeks of intervention thus including the interval in which clinical improvement has been reported in trials with patients with MD [28]. Personality variables were assessed with the NEO-PI-R and EPQ, both widely used self-report measures based on the Five-Factor Model of Personality [29].

Despite these advantages our trial also has a number of potential limitations. Firstly, a large number of women were excluded from our trial due to oral contraceptives and pregnancy, thus the trial population is characterized by an overrepresentation of men. Secondly, we cannot exclude that the dosage of 10 mg escitalopram was too low although this has been suggested as the optimum dose for treatment of moderate depression [30]. Even though the participants received weekly phone calls to optimise adherence, several of the participants in the escitalopram group were found to have low plasma escitalopram concentrations. We considered using a higher dosage, but escitalopram 20 mg daily might have given more adverse effects, eventually jeopardizing blinding and adherence, thus we decided to use 10 mg daily. This dose of escitalopram resulted in well-known adverse effects, such as sexual adverse effects, as described in a prior paper from the study [18]. Further, the exclusion of the two participants with immeasurable concentrations did not change the results substantially. Thirdly, it may be argued that four weeks of treatment is too short a period to affect aspects of personality. However, two prior studies on the effect of SSRI on personality revealed positive effects within a four week [8] and a two week [9] treatment period, respectively. Thus, these trials reported changes in relatively complex tasks or in specific measures (hostility, submissiveness) rather than the broad personality self-report descriptors as the NEO-PI or EPQ. Instead neuroticism was found to be affected after 8 weeks of administration by Tang et al. 2009. Moreover, we have not compared healthy individuals with a family history of MDD to healthy individuals without a family history of MD. Finally considering the multitude of statistical significance tests performed a p value of 0.046 may very well occur by chance and correction for multiple testing would render it insignificant. It should be noted that when adjusting for multiple analyses the p value for agreeableness was p = 0.28 using Holm's test. Thus the result needs to be confirmed in additional studies.

We planned to include 80 participants due to resources and availability of the healthy first-degree relatives of patients with MD, as previously described [17] and the AGENDA trial is the largest trial (n = 80) on healthy regarding any outcome, as shown in a recent review from our group [16]. We found a tendency for escitalopram to reduce neuroticism when measured by the NEO-PI-R, but the opposite tendency when neuroticism was measured by the EPQ. Thus, it may not seem likely that our results are due to type II errors and that a larger sample would have changed the results. Furthermore, neuroticism reported by EPQ and NEO-PI-R was closely correlated. It was not validated that the probands and the participants of the trial were indeed related. Furthermore, while our participants are at increased risk at depression many of them will not develop depression and the possible effect we found on agreeableness and conscientiousness may not be related to depression.

Results from a recent placebo-controlled trial in patients with major depression suggest that the SSRI paroxetine has a specific effect on personality traits of neuroticism and extraversion that is distinct from its effect on depression [4]. In a randomised controlled trial of patients with depression, neurotiscism and conscientiousness were significantly associated with response to a combination of medication and psychoterapy [31]. Our results suggest that escitalopram has no major direct effect on neuroticism, i.e., independently of the effect of depressive symptoms. We have chosen to recruit a sample of individuals with positive family history for MD, based on previous reports of increased neuroticism in this population. However, based on the mean scores reported in Table 1, the study sample presents with low-medium neuroticism levels thus breaking the original study design assumption. In fact, none of the subjects seem to report levels of neuroticism that would be considered as high and thus represent a risk factor for psychopathology. As familiarity for MD and neuroticism are likely to present both shared and non-shared contributions to higher vulnerability, it remains open for speculation whether a sample with positive family history but low-medium neuroticism scores (as in this trial) could in fact present some resilience towards the development of depression and perhaps a different response to antidepressants. This could also account for the negative results reported of 4 weeks antidepressant medication not affecting neuroticism levels. It could be hypothesised that changes in neuroticism by antidepressant medication only occur in the presence of baseline high neuroticism (as in a paper by Tang et al., 2009 [4]). This would be also supported by recent data showing that 1 week of antidepressant administration is able to modify negative biases in emotional processing correlated to high neuroticism [32]. According to the cognitive neuropsychological model of antidepressant drug action [33], antidepressants may work by modifying emotion processing biases and thus in turn producing changes at a phenotypic level such as symptoms and possibly personality measures.

Agreeableness is less studied in relation to depression. In a study of depressed patients (n = 53) [34] agreeableness was not significantly affected by SSRI treatment by flouoxetine. However, a study by Eskelius et al. found small but statistically significant changes in all the Karolinska Scales of Personality except the impulsiveness scale after 24 weeks of treatment with sertraline or citalopram in depressed patients. The changes (2.3–12.4%) were in the direction of normalisation, i.e., decreases in the anxiety and aggression-related scales and increases in social desirability and socialisation [35].

Studies of healthy first-degree relatives offer an excellent opportunity to determine whether personality traits represent a premorbid risk factor for subsequent onset of mood disorders or whether personality deviances are a consequence of mood episodes [15]. Future studies may explore the suggested link between the serotonergic system and the personality trait agreeableness. If the finding of changes in agreeableness is replicated, it may lead to the hypothesis that SSRI do not directly modulate neuroticism but rather mediate a different self-perception captured by changes in the scores of the facets of the personality dimension of agreeableness, which are trust, straightforwardness, altruism, compliance, modesty, and tender mindedness. Further it could be hypothesized that antidepressants first produce early changes in personality dimensions related to interpersonal measures in the direction of more positive interactions and that longer treatment is needed to affect self-reported measures of negative emotionality.

In conclusion, the AGENDA trial is the first trial to investigate if treatment with a SSRI has an effect on personality traits in healthy first-degree relatives of patients with major depressive disorder. No significant changes were found on any measure of neuroticism. In post-hoc analyses, escitalopram seemed to have an effect on agreeableness. Further, a trend was found for increased conscientiousness. Thus it is possible that SSRIs may have an effect on aspects of personality such as agreeableness and conscientiousness independent of the treatment effect on depression. The finding should be explored in future studies.

## Supporting Information

Checklist S1 CONSORT checklist.
(DOC)

Protocol S1 Trial protocol.
(DOC)
