# Supplementary material for: Effect of Chronic Escitalopram versus Placebo on Personality Traits in Healthy First-Degree Relatives of Patients with Depression: A Randomized Trial
Source: PLoS One. 2012 Feb 29;7(2):e31980. doi: 10.1371/journal.pone.0031980 (PMC3290539; doi:10.1371/journal.pone.0031980)
Supplement: Protocol S1 — Trial protocol. (DOC) [file pone.0031980.s002.doc]

# Trial Protocol SI

**FORSØGSPROTOKOL**

**FOR**

AGENDA

Associationer mellem genpolymorfier, endofænotyper for depression og antidepressiv behandling

Effekten af escitalopram versus placebo på raske med disposition for depression.

Et randomiseret, kontrolleret forsøg

**28. april 2008, version 6 (med 3. tillæg)**

Indholdsfortegnelse

Forside

[1. Generelle oplysninger 4](#__RefHeading___Toc153785609)

[2. Projektbeskrivelse 7](#__RefHeading___Toc153785610)

[**2.1. Resumé** 7](#__RefHeading___Toc153785611)

[**2.2. Flowdiagram** 8](#__RefHeading___Toc153785612)

[3. Titel 9](#__RefHeading___Toc153785614)

[4. Formål og hypoteser 9](#__RefHeading___Toc153785615)

[5. Litteraturgennemgang 9](#__RefHeading___Toc153785616)

[**5.1. Endofænotyper 9**](#__RefHeading___Toc153785617)

[**5.2. Serotonintransporteren** 10](#__RefHeading___Toc153785618)

[**5.3. Neuroendokrin dysregulation** 10](#__RefHeading___Toc153785619)

[**5.4. Personlighedstrækket neuroticisme** 10](#__RefHeading___Toc153785620)

[**5.5. Kognitiv dysfunktion** 10](#__RefHeading___Toc153785621)

[**5.6. Søvn** 11](#__RefHeading___Toc153785622)

[**5.7. Glucocorticoidreceptoren** 11](#__RefHeading___Toc153785623)

[**5.8. PET** 11](#__RefHeading___Toc153785624)

[**5.9. Forekomst af endofænotyper** 11](#__RefHeading___Toc153785625)

[**5.10. Medicinsk behandling af endofænotyper** 11](#__RefHeading___Toc153785626)

[6. Metode 12](#__RefHeading___Toc153785627)

[**6.1. Rekruttering af forsøgsdeltagere** 12](#__RefHeading___Toc153785628)

[**6.2. Inklusionskriterier** 13](#__RefHeading___Toc153785629)

[**6.3. Eksklusionskriterier** 13](#__RefHeading___Toc153785630)

[**6.4. Undersøgelsesprogram** 14](#__RefHeading___Toc153785631)

[6.4.1. Interview 14](#__RefHeading___Toc153785632)

[6.4.2. Spørgeskemaer 14](#__RefHeading___Toc153785633)

[6.4.3. Søvn 14](#__RefHeading___Toc153785634)

[6.4.4. Blodprøver 14](#__RefHeading___Toc153785635)

[6.4.5. Genetiske analyser 15](#__RefHeading___Toc153785636)

[6.4.6. Neuroendokrine undersøgelser 15](#__RefHeading___Toc153785637)

[6.4.7. Ekspressionsanalyser 15](#__RefHeading___Toc153785638)

[6.4.8. PET og MR-skanning 15](#__RefHeading___Toc153785639)

[6.4.9. Oversigt over forløbet af undersøgelser 15](#__RefHeading___Toc153785640)

[Undersøgelsesdag 1 15](#__RefHeading___Toc153785641)

[Undersøgelsesdag 2 15](#__RefHeading___Toc153785642)

[Undersøgelsesdag 3 16](#__RefHeading___Toc153785643)

[Undersøgelsesdag 4 16](#__RefHeading___Toc153785644)

[**6.5. Randomisering** 16](#__RefHeading___Toc153785645)

[**6.6. Medicin og placebo** 16](#__RefHeading___Toc153785646)

[**6.7. Ulemper, bivirkninger, risici og fordele for forsøgsdeltagerne** 17](#__RefHeading___Toc153785647)

[**6.8. Bivirkningsregistrering** 19](#__RefHeading___Toc153785648)

[**6.9. Rapportering af hændelser og bivirkninger** 20](#__RefHeading___Toc153785649)

[**6.10. Opfølgning ved hændelser eller bivirkninger** 20](#__RefHeading___Toc153785650)

[**6.11. Kriterier for forsøgs-/behandlingsstop** 20](#__RefHeading___Toc153785651)

[**6.12. Forsøgspersoner der ønsker at udgå af AGENDA (drop-outs)** 21](#__RefHeading___Toc153785652)

[**6.13. Opfølgning i forsøgsperioden** 21](#__RefHeading___Toc153785653)

[**6.14. Effektmål, materialestørrelsesberegning og statistiske analyser.** 21](#__RefHeading___Toc153785654)

[**6.15. Data-management.** 22](#__RefHeading___Toc153785655)

[**6.16. Procedure for brydning af kode til randomisering (emergency-break).** 22](#__RefHeading___Toc153785656)

[**6.17. Uafhængig datamonitorerings og sikkerhedskomite.** 23](#__RefHeading___Toc153785657)

[**6.18. Monitorering i henhold til reglerne om Good Clinical Practice (GCP).** 23](#__RefHeading___Toc153785658)

[7. Fremtidige undersøgelser 23](#__RefHeading___Toc153785659)

[8. Tidsplan 23](#__RefHeading___Toc153785660)

[9. Rapportering 24](#__RefHeading___Toc153785661)

[10. Interessekonflikter 24](#__RefHeading___Toc153785662)

[11. Etiske overvejelser 24](#__RefHeading___Toc153785663)

[12. Deltagerinformation 26](#__RefHeading___Toc153785664)

[13. Referencer 30](#__RefHeading___Toc153785665)

[Biobanken 37](#__RefHeading___Toc153785666)

[Center for Pharmacogenomics 37](#__RefHeading___Toc153785667)

[Sarstedt salivette kit,. 39](#__RefHeading___Toc153785668)

[**Teoretisk baggrund 40**](#__RefHeading___Toc153785669)

[**Kontraindikation og forsigtighedsregler 40**](#__RefHeading___Toc153785670)

[**Forberedelser 40**](#__RefHeading___Toc153785671)

[**Procedure 41**](#__RefHeading___Toc153785672)

**AGENDA vil blive gennemført efter denne forsøgsprotokol**

**22. februar 2007**

**Ulla Knorr Lars V. Kessing**

# 1. Generelle oplysninger

###### Sponsor, Investigator og GCP-ansvarlig

Ulla Knorr, 1. reservelæge, klinisk assistent, Psykiatrisk Klinik, Rigshospitalet, Blegdamsvej 9, 2100 Kbh. Ø. Tlf. 35456235.

Email: ulla.knorr@rh.regionh.dk

###### Hovedvejleder og Investigator

Lars V. Kessing, professor, dr. med., overlæge, Psykiatrisk Klinik, Rigshospitalet, Blegdamsvej 9, 2100 Kbh. Ø. Tlf. 35456177.

Email: [lars.kessing@rh.regionh.dk](mailto:lars.kessing@rh.regionh.dk)

###### Samarbejdspartnere

Ulrik Gether, professor, dr. med., Farmakologisk Institut, Panum Instituttet, Blegdamsvej 3, 2200 Kbh. N. Tlf. 35327602.

Email: [gether@neuropharm.dk](mailto:gether@neuropharm.dk)

Maj Vinberg, læge, ph.d., Psykiatrisk Klinik, Rigshospitalet, Blegdamsvej 9, 2100 Kbh. Ø.

Tlf. 35456235.

Email: maj.vinberg@rh.regionh.dk

Ulla Feldt - Rasmussen, klinikchef, dr. med., overlæge, Endokrinologisk Klinik, Rigshospitalet, Blegdamsvej 9, 2100 Kbh. Ø. Tlf. 35452337.

Email: [ufeldt@rh.dk](mailto:ufeldt@rh.dk)

Allan Hansen, bioanalytiker, Psykiatrisk Klinik, Rigshospitalet, Blegdamsvej 9, 2100 Kbh. Ø.

Tlf. 35456234.

Email: [allan.hansen@rh.dk](mailto:ah@rh.dk)

Gunnhild Helmsdal,

Gitte Moos Knudsen, professor, Neurobiologisk Forskningsenhed,

Rigshospitalet, Blegdamsvej 9, 2100 Kbh. Ø. Tlf. 35456712.

Email: [gitte@nru.dk](mailto:gitte@nru.dk)

Lisbeth Marner, klinisk assistent, Neurobiologisk Forskningsenhed,

Rigshospitalet, Blegdamsvej 9, 2100 Kbh. Ø. Tlf. 35456742.

Email: [lmarner@nru.dk](mailto:lmarner@nru.dk)

Anders Gade, lektor, mag. art., Institut for Psykologi, Københavns Universitet, Øster Farimagsgade 5A, 24., lok. 22, 1353 Kbh. K. Tlf. 35324880.

Email: [anders.gade@psy.ku.dk](mailto:anders.gade@psy.ku.dk)

Eva Meldal, stud. psych., forskningsassistent, Institut for Psykologi, Københavns Universitet, Øster Farimagsgade 5A, 24.0.1, 1353 Kbh. K. Tlf. 35324881.

Email: [eva.meldal@psy.ku.dk](mailto:eva.meldal@psy.ku.dk)

Helene Carstensen, stud. psych., forskningsassistent, Institut for Psykologi, Københavns Universitet, Øster Farimagsgade 5A, 24.0.1, 1353 Kbh. K. Tlf. 35324881.

Email: [helene.carstensen@psy.ku.dk](mailto:helene.carstensen@psy.ku.dk)

Eva Haastrup, læge, klinisk immunologi, H:S Blodbank,

Rigshospitalet, Blegdamsvej 9, 2100 Kbh. Ø. Tlf. 35453480.

Email: [rh13966@rh.dk](mailto:rh13966@rh.dk)

Henrik Ullum, overlæge, klinisk immunologi, H:S Blodbank,

Rigshospitalet, Blegdamsvej 9, 2100 Kbh. Ø. Tlf. 35453451.

Email: [rh02632@rh.dk](mailto:rh02632@rh.dk)

Christian Gluud, overlæge, dr. med., Copenhagen Trial Unit (CTU), Center for klinisk interventionsforskning, Panum Instituttet, 33-4-D, Blegdamsvej 3B, 2200 Kbh. N. Tlf. 35457175.

Email: [cgluud@ctu.rh.dk](mailto:cgluud@ctu.rh.dk)

Jørn Wetterslev, overlæge, ph.d., Copenhagen Trial Unit (CTU), Center for klinisk interventionsforskning, Panum Instituttet, 33-4-D, Blegdamsvej 3B, 2200 Kbh. N. Tlf. 35457159.

Email: [wetterslev@ctu.rh.dk](mailto:wetterslev@ctu.rh.dk)

Danmarks IT-center for uddannelse og forskning (UNI-C), Vermundsgade 5, 2100 Kbh. Ø.

Tlf. 35878889.

Email: [uni-c@uni-c.dk](mailto:uni-c@uni-c.dk)

GCP-enheden, Gentofte Amtssygehus, Niels Andersensvej 65, 2900 Hellerup.

Kontaktperson: Kristian Juul. Tlf. 39777417.

Email: [krijuu01@gentoftehosp.kbhamt.dk](mailto:krijuul@gentoftehosp.kbhamt.dk)

H. Lundbeck A/S, Otilliavej 9, 2500 Valby.

Kontaktperson: Læge, ph.d., Jan Egebjerg. Tlf. 36301311.

E-mail: [jege@lundbeck.com](mailto:jege@lundbeck.com)

H: S Apoteket, afsnit 4501,

Rigshospitalet, Blegdamsvej 9, 2100 Kbh. Ø. Tlf.: 35454501. Fax: 35456824.

###### Sikkerhedskomité

Per Bech, professor, dr.med., overlæge,

Psykiatrisk Forskningsenhed, Psykiatrisk Sygehus, 3400 Hillerød. Tlf. 48293253.

Email: [pebe@fa.dk](mailto:pebe@fa.dk)

Jørgen Hilden, lektor, læge, Biostatistisk Afdeling, Øster Farimagsgade 5, opg. B, Postbox 2099, 1014 Kbh. K. Tlf. 35327917.

Email: [j.hilden@biostat.ku.dk](mailto:j.hilden@biostat.ku.dk)

###### Leverandører

Nomeco A/S, Borgmester Christiansens Gade 40, 1790 Kbh. V.

Kontaktperson: Tina Holstein Hansen. Tlf. 36454536.

Email: [thh@nomeco.dk](mailto:thh@nomeco.dk)

NordMedica A/S, Bredgade 41, 1260 København K.

Tlf. 33337633.

Kontaktperson: Bent Andersen.

Email: [ba@nordmedica.com](mailto:ba@nordmedica.com)

Hounisens Laboratorieudstyr A/S, Sindalsvej 27, DK-8240 Risskov. Tlf. 86210800.

Email: [hounisen@mail.dk](mailto:hounisen@mail.dk)

###### Journalnumre

Videnskabsetisk Komite, AGENDA: KF 02-307413 med amendments KF 12 2006-4542 og H-KF-307413.

Videnskabsetisk Komite, Dansk Psykiatrisk Biobank: KF 01-024/01.

Lægemiddelstyrelsen: 2612-3162.

EudraCT: 2006-001750-28.

Datatilsynet: 2006-41-6737.

ClinicalTrials.gov: NCT 00386841

# 2. Projektbeskrivelse

## **2.1. Resumé**

Der stilles i stigende grad spørgsmål ved, om moderne diagnostiske kriterier for depression er fyldestgørende, dvs. hvorvidt de afgrænser depression som en sygdomsenhed (eller en sand fænotype). Derfor har forskning i depression i de seneste år i stigende grad fokuseret på udforskning af endofænotyper. Endofænotyper er arvelige biologiske eller psykologiske markører som forekommer med øget hyppighed hos patienter med depression og hos raske slægtninge til patienter med depression. Tidligere studier peger på, at mulige endofænotyper for depression er personlighedstrækket neuroticisme, diskrete indlærings- og hukommelsesproblemer (kognitiv dysfunktion), øget stressrespons (dysregulation af hypothalamus-hypofyseforlap-binyrebark (HPA)-aksen) og specifikke hjerneforandringer (abnorm 5-HT4 receptor funktion).

Det er uvist, om antidepressiv medicinsk behandling virker ved at normalisere abnorme endofænotyper. Behandling med antidepressiva hos patienter med en akut depressiv episode er associeret med normalisering af endofænotyper, men i sådanne studier er det ikke muligt at skelne effekten på endofænotyperne fra effekten på selve depressionen, da remission af depressive symptomer er associeret med delvis normalisering af endofænotyperne. For at undersøge effekten af antidepressiv medicin på endofænotyper er det nødvendigt at undersøge en gruppe personer, som frembyder endofænotyperne, men ikke er syge. Raske første-gradsslægtninge til patienter, som lider af depression, udgør sådanne personer.

I aktuelle studie af raske personer med arvelig disposition for depression undersøges hvorvidt mulige endofænotyper 1) er associeret med specifikke genpolymorfier 2) nedarves fra patienter med depression til deres børn 3) påvirkes af behandling med serotonin-genoptagelseshæmmere (ssri).

Undersøgelsen er et 4-ugers varende randomiseret, dobbeltblindt studie, hvor deltagerne deles i to grupper, som behandles med enten antidepressiv medicin (tablet Cipralex® 10 mg) eller placebo. Der forventes inklusion af 80 raske forsøgspersoner, som er disponeret for udvikling af depression, idet de har en forældre med nylig behandlet depressiv lidelse.

Forsøgspersonerne identificeres i et igangværende studie under Center for Pharmacogenomics, Københavns Universitet, som voksne børn **eller søskende** af 500 patienter, som ifølge det Danske Psykiatriske Centrale Forskningsregister for nyligt er blevet behandlet for depression ved en psykiatrisk afdeling på Sjælland. Ca. 200 personer vil blive inviteret og deltagerprocenten forventes at blive ca. 40 %.

Forsøgspersonerne bliver før og efter 4 ugers behandling grundigt interviewet vedrørende psykiatriske symptomer, herunder depressive symptomer, personlighedstræk, forekomst af stressende begivenheder, oplevet stress og kognitiv funktion. Effekten af antidepressiv medicin på stress niveauet registreres ved måling af spyt-cortisol og ved respons på den kombinerede dexamethason corticotropin-releasing hormontest. I et parallelt udføres tillige udføres MR og PET skanning af 5-HT4 receptor funktionen før og efter 4 ugers behandling (der forventes gennemført skanninger på i alt 40 personer).

Projektet udføres som et 3-årigt ph.d.-projekt af 1. reservelæge, klinisk assistent Ulla Knorr, Psykiatrisk Klinik, Rigshospitalet, som er ansat per 1/2-2006 for midler fra Københavns Universitet.

## **2.2. Flowdiagram**

# Forsøgsoversigt

Danske forsøgspersoner i alderen 18 – 60 år med arvelig disposition for depression

Informeret samtykke

RANDOMISERING

Dobbelt

blindt

**Undersøgelses-**

**program**

**Undersøgelses-**

**program**

**Cipralex per os i**

**28 dage**

**Placebo per os i**

**28 dage**

**Undersøgelses-**

**program**

**Undersøgelses-**

**program**

# 3. Titel

Associationer mellem genpolymorfier, endofænotyper for depression og antidepressiv behandling, (AGENDA)

# 4. Formål og hypoteser

Den overordnede hypotese er, at personlighedstrækket neuroticisme, kognitiv dysfunktion, dysregulation af hypothalamus-hypofyseforlap-binyrebark (HPA)-aksen og specifikke hjerneforandringer udgør endofænotyper for depression.

Følgende specifikke alternative hypoteser fremsættes:

Personlighedstrækket neuroticisme, kognitiv dysfunktion, dysregulation af HPA-aksen og specifikke hjerneforandringer (ændringer i 5-HT4 receptor systemet)

1. Er associeret med specifikke genpolymorfier.
2. Nedarves fra patienter med depression til deres børn.
3. Påvirkes af behandling med selektive serotonin reuptake inhibitorer (ssri).

Formålet er at teste ovenstående hypoteser, dog undersøges kun personlighedstrækket neuroticisme i hypotese 2.

# 5. Litteraturgennemgang

## **5.1. Endofænotyper**

I de seneste år er en ny strategi for genetisk forskning indenfor psykiatriske lidelser vundet frem. Sammenhænge mellem genotype og forskellige kvantitative komponenter ved fænotypen undersøges. Disse komponenter kaldes endofænotyper og de kvantificeres med neurofysiologiske, biokemiske, endokrine, neuroanatomiske, kognitive eller neuropsykologiske metoder [1]. Endofænotyper udgør en undergruppe af markører eller biomarkører, men adskiller sig fra disse ved at opfylde specifikke kriterier. Endofænotyper er: Associeret med sygdom i befolkningen, er arvelige, kan være aldersafhængige, men forekommer stabilt over tid, og de påvises eventuelt ved stimulationstest. Endofænotyper og den undersøgte sygdom co-segregerer i familier og de forekommer hyppigere hos syge og deres raske slægtninge end i baggrundsbefolkningen.

Endofænotyper underkastes på denne måde tilsvarende kriterier, som anvendes ved diagnostisk validering af psykiatriske lidelser [2] og et muligt fremtidsperspektiv er udvikling af forbedrede prækliniske modeller [3], hvor sygdomme klassificeres på grundlag af endofænotyper frem for specifikke nosologiske klasser [4] og via kliniske forskning er det håbet herved at opnå bedre diagnostik og behandling.

## **5.2. Serotonintransporteren**

Data fra prækliniske og farmakologiske studier vedrørende serotonintransporteren (5-HTT) støtter hypotesen om, at en dysfunktion i hjernens serotonerge systems aktivitet bidrager til øget sårbarhed for depressiv lidelse [5;6]. 5-HTT er det *major site* for serotonin reuptake i det præsynaptiske neuron. Genet for 5-HTT er lokaliseret på kromosom 17 på positionen 17q11.1 -17q12 [7;8] og en 44-basepar insertions/deletionspolymorfi i 5-HTT promotor regionen (5-HTTLPR), resulterer i en kort og en lang variant af genet, som fører til varierende transkriptionel aktivitet [9].

”Lang/kort” 5-HTTLPR-genpolymorfien er blevet undersøgt grundigt hos patienter med depressive tilstande, men resultaterne er modstridende. Det er uklart, om visse typer af 5-HTTLPR genpolymorfien er hyppigere forekommende hos patienter med depressive lidelser end hos baggrundsbefolkningen [10;11] og om de kan være markører for behandlingsrespons med ssri [12]. De modstridende resultater kan skyldes begrænsede undersøgelsespopulationer foruden patienter og kontrolpersoners genetiske heterogenitet.

Som det fremgår nedenfor, formodes det på baggrund af flere undersøgelser, at 5-HTTLPR genpolymorfien kunne være relateret til personlighedstrækket neuroticisme, kognitiv dysfunktion og dysregulation af det neuroendokrine system og at disse kunne være mulige endofænotyper for depressiv lidelse.

## **5.3. Neuroendokrin dysregulation**

Ved akut depression kan dysregulation af HPA-aksen påvises hos en stor del af patienterne [13]. Dysregulationen kan vise sig ved hyperaktivitet i form af forhøjet plasma- og spytcortisolniveau og nedsat evne til suppression i kombineret dexamethason corticotropin-releasing hormontest (DEX-CRH). Dysregulation af HPA-aksen kan tillige påvises hos patienter med depressiv lidelse i remissionsfasen og i mindre grad hos raske personer med familiær disposition for depressiv lidelse sammenlignet med raske uden familiær disposition [14-20]. Dette peger på, at dysregulation af HPA-aksen kan være en endofænotype for depressiv lidelse. Det er ikke undersøgt om 5-HTTLPR-genpolymorfier er relaterede til neuroendokrin dysregulation som endofænotype for depressiv lidelse.

## **5.4. Personlighedstrækket neuroticisme**

Personlighedstrækket neuroticisme beskrives udfra standardiserede spørgeskemaer, NEO-PI-R og Eysenck. Studier af raske, som ud fra deres familiehistorie genetisk set tilhører en højrisikogruppe for udvikling af depression, tyder på, at personlighedstrækket neuroticisme er en endofænotype for depressiv lidelse [21-24]. I et nyligt publiceret studie finder man, at personlighedstrækket neuroticisme kan forklare 42 % af effekten af 5-HTTLPR for livstidsrisikoen for at udvikle depressiv lidelse [25]. Materialet er dog talmæssigt begrænset.

## **5.5. Kognitiv dysfunktion**

Patienter med depression udviser en bred vifte af kognitive deficits [26] og en del af patienterne har kognitiv dysfunktion også når de er velbefindende og i euthyme faser af sygdommen [27-33]. Et højrisiko-studium fra Psykiatrisk Klinik, Rigshospitalet har vist diskrete kognitive forringelser hos raske personer med disposition til bipolar affektiv sindslidelse, [34]. Det er ikke undersøgt om 5-HTTLPR-genpolymorfier er relaterede til kognitiv dysfunktion, som en mulig endofænotype for depressiv lidelse.

## **5.6. Søvn**

Søvnforstyrrelser indgår som et symptom i depressionsdiagnosen og er foreslået som en mulig endofænotype for depression [35].

## **5.7. Glucocorticoidreceptoren**

På det molekylære niveau er mekanismerne for dysregulation af HPA-aksen ved depressiv lidelse uklare. Det er muligt, at dysfunktion af glucocorticoidreceptoren (GR), som spiller en vigtig rolle i den negative feedbackregulation af HPA-aksen og adaptation til stress, kan spille en central rolle. Binding af glucocorticoider til GR medfører inhibition af sekretion og syntese af både cortico-releasing hormon (CRH) og adrenocorticotropt hormon (ACTH). Foruden GRs rolle i HPA-aksen forekommer GR adskillige steder i hjernen, hvor receptoren menes at modulere forskellige neuronale funktioner såsom indlæring og hukommelse. GR har to protein isoformer, GR-α og GR-β. GR-α har glucocorticoid effekt. GR-β kan ikke binde glucocorticoider, men menes at indgå i regulationen af GR-α-medieret transkription. Flere undersøgelser har peget på, at GR spiller en vigtig rolle i patofysiologien ved depressive lidelser og i et netop publiceret studie [36], påvises reduceret ekspression af GR-α mRNA hos både patienter med depressiv og bipolar sindslidelse, såvel under en akut depressiv episode som i remissionsfasen. Hos førstegradsslægtninge af patienter med bipolar sindslidelse påvises tillige GR-α mRNA-reduktion.

## **5.8. PET**

I studier af patienter med unipolar depression findes nedsat volumen af hippocampus ved anvendelse af magnetisk resonans (MR) skanning [37] og med positron emission tomografi (PET) kan funktionelle abnormaliteter i hippocampus påvises hos samme patientgruppe [38;39]. Hos mennesker er høje koncentrationer af 5-HT4 receptorer fundet i putamen og nucleus caudatus [40]. Imidlertid er sammenhængen kun undersøgt i et enkelt humant studie, som også fandt en højere forekomst af 5-HT4 receptorer hos patienter med depression [41]. Det er ikke vist om specifikke hjerneforandringer påvirkes af escitalopram hos personer med arvelig disposition for depression.

## **5.9. Forekomst af endofænotyper**

Baseret på resultater fra tidligere studier [42], estimeres det, at der på enkeltpersonsniveau, hos 30 % af raske personer med arvelig disposition for depressiv lidelse, vil kunne påvises mindst to af de tre endofænotyper; personlighedstrækket neuroticisme, kognitiv dysfunktion eller dysfunktion af HPA-aksen. Forekomsten af øvrige mulige endofænotyper er uafklaret.

## **5.10. Medicinsk behandling af endofænotyper**

Ovennævnte studier har vist, at behandling med antidepressiva hos patienter med en akut depressiv episode er associeret med reduktion i personlighedstrækket neuroticisme, forbedring af kognitiv funktion og delvis normalisering af HPA-AKSEN aksen. I sådanne studier er det ikke muligt at skelne effekten på endofænotyperne fra effekten på selve depressionen, da remission af depressive symptomer er associeret med delvis normalisering af endofænotyperne. Det vides ikke om behandlingsrespons ved depression er medieret gennem en effekt på endofænotyperne.

Der foreligger resultater af nyere randomiserede forsøg, som giver formodning om, at behandling med et ssri præparat versus behandling med placebo påvirker basale personlighedstræk og adfærd hos personer uden psykisk sygdom [43-45]. Der er ikke tidligere foretaget kliniske forsøg af escitaloprams virkning på en veldefineret gruppe af raske førstegradsslægtninge til patienter, som lider af depression.

# 6. Metode

Forsøget er et 4-ugers varende randomiseret og placebokontrolleret studie af ssri (escitalopram 10 mg per dag) versus placebo, hvor forsøgsdeltagere, investigatorer, databehandlere og statistiker er blindet.

Forsøget udgør et delprojekt under Center for Pharmacogenomics: [www.genpharm.dk](http://www.genpharm.dk/).

Når alle relevante instanser har accepteret gennemførsel af AGENDA initieres forsøget ved udsendelse af et nyhedsbrev til samtlige samarbejdspartere, som samtidig orienteres om at de er forpligtigede til at følge protokollen. Nyhedsbrev vil blive udsendt, når halvdelen af forsøgspersonerne har gennemført forsøget og ved dets afslutning samt ved behov i øvrigt.

## **6.1. Rekruttering af forsøgsdeltagere**

I et igangværende projekt (KF 01-209/04) på Center for Pharmacogenomics, Københavns Universitet vil omkring 500 patienter, som har afsluttet første behandlingsforløb - under indlæggelse eller ambulant - ved en psykiatrisk afdeling på Sjælland med den kliniske diagnose depressiv enkeltepisode (DF32.0 - DF32.9) blive identificeret fortløbende fra det Danske Psykiatriske Centrale Forskningsregister. Patienterne bliver interviewet og diagnosticeret i henhold til Schedules for Clinical Assessment in Neuropsychiatry (SCAN) [46]. Patienter, hvor den kliniske diagnose bekræftes af forskningsdiagnosen opnået ved SCAN interview og patienter som får forskningsdiagnosen tilbagevendende depression F33.0 – 33.9, bliver bedt om at give tilsagn til, at deres eventuelle voksne søskende eller børn forespørges om ønske til deltagelse i aktuelle AGENDA.

**Herudover vil patienter, som ved indlæggelse eller ambulant behandling på Psykiatrisk Center Rigshospitalet, har fået diagnosen depression** **blive bedt om at give tilsagn til, at deres eventuelle voksne børn og søskende forespørges om ønske til deltagelse i aktuelle AGENDA**.

De voksne søskende eller børn vil blive kontaktet per brev med skriftlig information samt forespørgsel om deltagelse i ADENDA forsøget, hvilket Sponsor-Investigator registrerer i screeningslog. Hvis forsøgsdeltagere ikke responderer på første henvendelse, vil de blive kontaktet igen enten per brev eller telefonisk. Forsøgsdeltagerne informeres mundtligt om forsøget dels telefonisk dels ved fremmøde i rolige omgivelser på ansøgers kontor. I deltagerinformationen gøres opmærksom på muligheden for en bisidder. For at kunne indgå i forsøget, skal forsøgsdeltagerne opfylde alle følgende inklusionskriterier og ingen af eksklusionskriterierne.

Forsøgspersonerne oprettes ikke i sygehusets dokumentationssystem (”Det grønne system). CRF opfylder § 9 i lægers pligt til journalføring.

## **6.2. Inklusionskriterier**

- Voksne (18 – 60 år) med disposition for depression, rekrutteret som ovenfor beskrevet.

- Født i Danmark med europæiske forældre og bedsteforældre.

- For fertile kvinder sikres, at de ved inklusion ikke er gravide (negativ graviditetstest), samt at de gennem hele forsøget og indtil 6 dage ( 5 x t½ for Cipralex® = 5 x 30 timer => 6 dage) efter sidste indtagne tablet (Cipralex® eller placebo), anvender spiral eller dobbeltbarrieremetode (f.eks. kondom/ pessar eller kondom/sæddræbende creme). Kvinder vil blive informeret om, at graviditet skal undgås i forsøgsperioden, dels fordi det vil påvirke forsøgsresultaterne, dels fordi der er en, om end meget beskeden risiko, for fosterskader ved Cipralex®-behandling. Kun kvinder, som skønnes at kunne forstå denne information, kan inkluderes. Kvinder informeres om at sikker prævention skal anvendes i hele forsøgsperioden plus 6 dage. Graviditetsprøve vil blive gentaget på 3. undersøgelsesdag som kontrol.

- Afgivet informeret samtykke efter skriftlig og mundtlig information ved at underskrive samtykke og fuldmagtserklæring vedrørende AGENDA og Bio-bank.

## **6.3. Eksklusionskriterier**

- Somatisk sygdom eller andet handikap, som praktisk eller sprogligt gør deltagelse i forsøget umulig.

- Amning eller graviditet.

- Manglende informeret samtykke.

Ved testningen af hypotese 3 er eksklusionskriterierne yderligere skærpet og forsøgsdeltagere ekskluderes ved:

- Daglig indtag af naturlægemidler indeholdende Perikum (hypericum perforatum) eller medicin med kendt interaktion med escitalopram: MAO-hæmmere, perorale antikoagulantia eller lægemidler, der påvirker trombocytfunktionen; atypiske antipsykotika, fetiaziner, de fleste tricykliske antidepressiva, acetylsalicylsyre og non-steroide antiinflammatoriske lægemidler (NSAID), ticlopidin og dipyridamol. Endvidere lægemidler med serotonerg effekt såsom sumatripan eller andre tritaner, tramadol, tryptophan og præparater som er kendt for at forårsage hyponatriæmi; ssri, neuroleptika, fenatiaziner, thioxanthener, butyrofenoner, mefloquin, bupropion., litium, omeprazol, cimetidin, esomeprazol, fluvoxamin, lansoprazol, ticlopidin, cimetidin, flecainid, propafenon og metoprolol.

- Dagligt indtag af lægemidler der har interaktion med kortikosteroider; phenotoin, phenobarbital, efedrin, indomethacin, rifampin og orale kontraceptiva eller andre former for hormonel antikonceptiva (gestagendepotinjektion, subdermal implantation, hormonal vaginalring eller transdermal depotplaster)

- Overfølsomhed over for escitalopram, dexametason eller human corticotropin-releasing hormon samt produkternes øvrige indholdsstoffer.

- Tidligere medicinsk eller psykologisk behandling for lidelser i det skizofrene eller affektive spektrum.

- Pågående misbrug af psykoaktive stoffer.

Rapportering af forsøgspersonernes vej gennem forsøget beskrives i henhold til ”The Consort E-Flowchart” www.consort-statement.org.

## **6.4. Undersøgelsesprogram**

### *6.4.1. Interview*

*1. Basisoplysninger*: Fødselsvægt, højde, vægt, hofte- og abdominalomfang samt håndethed, Edinburgh Inventory [47].

*2. Diagnose*: Schedules for Clinical Assessment in Neuropsychiatry (SCAN).

*3. Depressive symptomer*: Hamilton Depression Scale HAM-D, 17-items [48] og Hamilton 14-item Angstskala [49].

*4. Personlighedsafvigelse*: The Structured Clinical Interview for DSM – IV Axis II Personality Disorders (SCID – II) [50].

*5. Kognition*: Kognitive funktioner testes med et batteri af neuropsykologiske test, der måler hukommelse, opmærksomhed, sproglige færdigheder og mobilisering, visuospatialt tempo, risikovillighed, kognitiv automatisering, logisk tænkning, genkendelse og forståelse af emotioner og socialkognitiv indsigt: Danish Adult Reading Test [51], Kendte ansigter (29 stk.) [52], Trail Making A and B [53], Stroop test [54;55], Boston naming [56], Blokmønsterprøven [57], Category Cued Recall [58], Cambridge Cognitive Examination (CAMCOG) [59], Rey Auditory Learning Test [60], Rey complex figure [61], ordmobilisering [62], SDMT [63], Iowa Gambling Task [64], Letter-number-sequencing [65], Social translations [66], moralsk adfærd [67], moralske dilemmaer [68], MSCEIT [69] og emotionssekskanten [70].

Ved undersøgelserne assisterer Eva Meldal og Helene Carstensen under supervision af Anders Gade.

### *6.4.2. Spørgeskemaer*

*1. Personlighedstræk*: Eysenck Personality Questionnaire (EPQ) [71], NEO-PI-R [72], Standardised Assessment of Personality – Abbreviated Scale (SAPAS) [73], Buss-Perry Aggression Quistionnaire [74] og SCID-II –personlighedsspørgeskema.

*2. Aktuelle depressionsgrad*: Beck Depression Inventory, 42-items (BDI) [75;76].

*3. Bivirkninger*: Side Effect Self Rating Scale (UKU-SERS-Pat) [77].

*4. Livskvalitet*: Quality of Life (WHOQOL– BREF) [78].

*5. Oplevet stress*: Perceived Stress Scale [79].

*6. Livshændelser*: Kendlers questionnaire for lifetime events [80], forkortet dansk udgave.

*7. Smerteregistrering:* Visuel Analog Skal modificeret efter Klepstad [81].

### *6.4.3. Søvn*

*Søvn* opgøres før og efter 4 ugers behandling med HAM-D foruden tillægsspørgsmål om antal episoder med afbrudt søvn, samlet antal nattesøvntimer samt subjektiv søvnkvalitet over de sidste tre døgn med en visuel analogskala (VAS).

### *6.4.4. Blodprøver*

*Veneblod* udtages til generel helbredsundersøgelse inkl. elektrolytter, hæmoglobin, blodsukker, levertal, nyrefunktion, thyroideatal, lipider, cholesterol og eventuel graviditetstest. Herudover udtages veneblod til måling af immunologiske parametre, genanalyser, spyt/plasma-cortisol, plasma-ACTH, mRNA oprensning, serum-escitalopram og Bio-bank. Alle blodprøverne er rutinemæssige prøver/analyser. Der føres optegnelser over prøver i Psykiatrisk Biobank og opbevarede prøver, som direkte indgår i forsøget til senere analyse, bilag 1 og 2.

### *6.4.5. Genetiske analyser*

*Genetiske undersøgelser* af bl.a. polymorfier i serotonintransporteren og catechol-O-methyltransferase (COMT) mv. udføres af Center for Pharmacogenomics. Blodprøverne indgår ligeledes i Dansk Psykiatrisk Biobank mhp. fremtidige genanalyser (Journalnr. KF 01-024/01).

### *6.4.6. Neuroendokrine undersøgelser*

*Kombineret dexamethason corticotropin-releasing hormontest:* Udføres i henhold til internationale standarder [82]: På 1. undersøgelsesdag kl. 23.00 gives 1,5 mg dexamethason per os. Den efterfølgende dag kl. 14.00 lægges venflon i en antecubital vene. Forsøgsdeltageren hviler i rygleje i mindst 30 minutter, og kl.15.00 gives 100 ug human CRH gives intravenøst. Til bestemmelse af plasma-cortisol og plasma-ACTH tages blodprøver tages hvert kvarter fra kl. 14.00 til kl. 18.00.

På samme tidspunkter måles spytcortisol.

Ved undersøgelserne assisterer Allan Hansen.

### *6.4.7. Ekspressionsanalyser*

Ekspressions-analyser foretages på veneblod i samarbejde med H. Lundbeck A/S.

### *6.4.8. PET og MR-skanning*

En del af deltagerne (ca. 40) vil blive spurgt, om de vil deltage i MR og PET skanning med receptor undersøgelse af hjernens funktion (protokol anmeldes særskilt). Denne del af forsøget udføres i Neurobiologisk forskningsenhed, Rigshospitalet og på Billeddiagnostisk afd. Hvidovre Hospital.

Ved MR-skanning anvendes ikke kontrast.

### *6.4.9. Oversigt over forløbet af undersøgelser*

### Undersøgelsesdag 1

Mundtlig information.

Underskrivelse af samtykke- og fuldmagtserklæringer.

Basisoplysninger inkl. bankoplysninger.

Interview om fysisk og psykisk helbred.

In- eller eksklusion.

Spørgeskemaer.

PET information.

Oplysning om videre forløb.

Randomisering og udlevering af forsøgsmedicin og CRH.

Blodprøver til genanalyser, Dansk Psykiatrisk Biobank og almindelig helbredsundersøgelse.

Spytprøve.

Frokost-pause.

Neuropsykologisk undersøgelse.

### Undersøgelsesdag 2

Hormonresponstest inkl. spytcortisol-undersøgelser.

Herefter daglig indtagelse af forsøgsmedicin i 28 dage.

### Undersøgelsesdag 3

Interview.

Spørgeskemaer.

Blodprøver.

Neuropsykologisk undersøgelse.

### Undersøgelsesdag 4

Hormonresponstest inkl. spytcortisolundersøgelser. Lægesamtale med aftale om aftrapning af forsøgsmedicinen med ½ tablet dagligt i 5 dage.

Forsøgspersonerne opsamler spyt til måling af cortisol hjemme før undersøgelsesdagene 2 og 4.

## **6.5. Randomisering**

Randomisering forestås af Copenhagen Trial Unit (CTU), Center for klinisk interventionsforskning, Rigshospitalet. Der foretages stratificering på køn og alder. Randomiserings- og randomiseringskoden opbevares i CTU. Følgende procedure følges: H. Lundbeck A/S udarbejder en liste med oplysning om indholdet (interventionsmedicin eller placebo) af medicinpakninger nummereret 100-250. Listen leveres CTU, som udarbejder en passende blokrandomisering. Når en forsøgsperson skal randomiseres foregår det ved opringning af Ulla Knorr eller Lars V. Kessing til CTU på tlf. 35457171; mandag til torsdag 9 – 16 og fredag 9 – 14.

## **6.6. Medicin og placebo**

###### Modtagelse af medicin

Forsøgsmedicin, Cipralex®, escitalopram 10 mg eller placebo leveres af H. Lundbeck A/S i identiske pakninger med identisk udseende kapsler indeholdende enten Cipralex® eller placebo, som er en uvirksom kalktablet, som hverken ved lugt, smag, opløselighed eller farve kan adskilles fra Cipralex®. Forsøgsmedicinen administreres dagligt som en tablet ved sengetid og dosisjustering tillades ikke. Efter 4 ugers indtagelse af Cipralex® /placebo foretages nedtrapning med Cipralex® 5 mg/placebo over 5 dage.

Vedlagt som bilag findes forenklet Investigational Medicinal Product Dossier (IMPD) for placebo og fremstillertilladelse fra H. Lundbeck A/S.

Lægemidlet tablet Decadron®, dexamethason 0,5 mg er ikke indregistreret i Danmark. Udleveringstilladelse søges fra Lægemiddelstyrelsen. Decadron® bestilles fra NOMECO A/S. Såfremt tilladelsen modtages sendes den til H:S Apotekets ekspeditionsafdeling og herefter kan Decadron® bestilles fra H:S Apoteket pr. telefon og FAX.

Injektionssubstans human corticotropin-releasing hormon (Cortirel® 100 ug) er ligeledes ikke indregistreret i Danmark. Udleveringstilladelse søges fra Lægemiddelstyrelsen. Curatis er producenten, importøren er NordMedica, som via Nomeco leverer Cortirel® via H:S Apoteket.

Medicinregnskab føres for forsøgsmedicinen; Cipralex®, placebo, og Decadron®: Medicin id (batch nr. kode), mængde udleveret (antal tabletter/kapsler), hvem medicinen udleveres til (forsøgsdeltager løbenr.), hvem medicinen udleveres af (dato og initialer), mængde returneret til destruktion (antal pakninger/tabletter/kapsler) og hvem medicinen afleveres til (dato og initialer).

For Cortirel® føres regnskab over: Medicin id (batch nr. kode), mængde administreret (antal ug), hvem medicinen administreres til (forsøgsdeltager løbenr.) og hvem medicinen administreres af (dato og initialer).

###### Håndtering af medicin

Fra H:S Apoteket og H. Lundbeck A/S leveres medicinen til afsnit 6234, Rigshospitalet. Etikettering varetages af H:S Apoteket.

###### Opbevaring af medicin

Cortirel® skal opbevares i fryser ved temperaturer mellem –20 og -10 grader, dog kan det opbevares ude af fryser i 48 timer. Der er ingen særlige forsigtighedsregler vedrørende opbevaring af Decadron® og Cipralex®..

Cortirel® transporteres fra H:S Apoteket til fryser i Neuropsykiatrisk laboratorium i frysebox. Forsendelseskvitteringer fremsendes til sponsor/investigator. I affektiv forskningsenhed opbevares Decadron® og Cipralex® på sponsor/investigators kontor i aflåst arkivskab.

###### Destruktion af medicin

H:S Apoteket modtager ikke anvendt medicin, eller medicin hvor holdbarhedsdatoen er overskredet, til destruktion.

## **6.7. Ulemper, bivirkninger, risici og fordele for forsøgsdeltagerne**

*Ulemper*

Der er en ulempe ved – på grund af forælderens depression – at blive inddraget i forsøget og mindet om egen sygdomsrisiko. Der er en tidsmæssig ulempe ved interview og udfyldelse af spørgeskemaer. Der er beskedent ubehag ved blodprøvetagning og anlæggelse af venflon i en vene. Endvidere kan bivirkninger ved de givne lægemidler forekomme.

###### Bivirkninger

Bivirkninger til Decadron® givet som engangsdosis som anført i punkt 6.5.7. er kortvarige og reversible. Det drejer sig om rastløshed, søvnproblemer, psykisk påvirkning, højt blodsukker og forhøjet blodtryk.

Ved Cortirel® forekommer lettere bivirkningerne hos 20% og sjældent optræder generende rødmen og varmefornemmelse af ca. ½ times varighed.

Der er ikke kontraindikationer eller særlige forsigtighedsregler ved indgift af CRH.

Af Lægemiddelstyrelsens produktresumé for Cipralex® (escitalopram) fremgår følgende:

Bivirkninger forekommer hyppigst i løbet af den første eller anden uge af behandlingen og aftager normalt i intensitet og hyppighed ved fortsat behandling. Efter langvarig behandling kan et pludseligt ophør med ssri forårsage seponeringssymptomer hos nogle patienter. Selvom disse seponeringssymptomer kan forekomme ved seponering af behandling, tyder de prækliniske og kliniske data ikke på, at ssri giver anledning til afhængighed. Seponeringssymptomer (svimmelhed, hovedpine og kvalme) er observeret hos nogle patienter efter brat seponering af behandling med escitalopram. De fleste symptomer var milde og forbigående. For at undgå seponeringssymptomer anbefales gradvis seponering over 1-2 uger. I dobbeltblinde, placebokontrollerede undersøgelser forekom følgende bivirkninger hyppigere under behandling med escitalopram end med placebo. Nedenstående hyppigheder er ikke placebokorrigerede.

| Metaboliske og ernæringsmæssige lidelser | Almindelige ** | Nedsat appetit |
| --- | --- | --- |
| Psykiatriske lidelser | Almindelige ** | Kvinder og mænd: Nedsat libido. Kvinder: Anorgasmi |
| Lidelser i centralnervesystemet | Almindelige ** | Insomnia, somnolens, svimmelhed |
|  | Sjældne *** | Smagsforstyrrelser, søvnforstyrrelser |
| Respiratoriske lidelser, lidelser i thorax og mediastinum | Almindelige ** | Sinusitis, gaben |
| Gastrointestinale lidelser | Meget almindelige * | Kvalme |
|  | Almindelige ** | Diarré, obstipation |
| Dermatologiske lidelser | Almindelige ** | Øget svedtendens |
| Forstyrrelser i reproduktion og lidelser i mammae | Almindelige ** | Mænd:  Ejakulationsforstyrrelser, impotens |
| Generelle lidelser og forstyrrelser ved applikationssted | Almindelige ** | Træthed, feber |

* (>1/10) ** (>1/100, <1/10) *** (>1/1000, <1/100)

Følgende bivirkninger er set ved behandling med ssri generelt:

| Metaboliske og ernæringsmæssige lidelser | Hyponatriæmi, utilstrækkelig sekretion af ADH |
| --- | --- |
| Psykiatriske lidelser | Hallucinationer, mani, konfusion, agitation, angst, depersonalisation, panikanfald, nervøsitet |
| Lidelser i centralnervesystemet | Kramper, tremor, koordinationsforstyrrelser, serotonergt syndrom |
| Lidelser i øjne | Synsforstyrrelser |
| Kardiovaskulære lidelser | Ortostatisk hypotension |
| Gastrointestinale lidelser | Kvalme, opkastning, mundtørhed, diarré, anoreksi |
| Leversygdomme | Forhøjede leverprøver |
| Dermatologiske lidelser | Udslæt, ekkymose, kløe, angioødem, svedtendens |
| Muskeloskeletale og bindevævslidelser | Ledsmerter, muskelsmerter |
| Lidelser i nyre- og urinveje | Urinretention |
| Forstyrrelser i reproduktion og lidelser i mammae | Galaktoré, seksuel dysfunktion inkl. impotens, forsinket sædafgang, anorgasmi |
| Generelle lidelser og forstyrrelser ved applikationssted | Insomnia, svimmelhed, træthed, sløvhed, anafylaktisk reaktion |

Serotonergt syndrom kan forekomme med en hyppighed på 0.3 promille af patienter indlagt med depression [83]. Det er omdiskuteret, om behandling med escitalopram i sjældne tilfælde kan udløse suicidale impulser eller eufori hos patienter, som lider af depression [84-87]. Der foreligger ikke meddelelser om udløsning af serotonergt syndrom, suicidale impulser eller eufori hos raske personer, som er behandlet med escitalopram. Disse bivirkninger må forventes at forekomme yderst sjældent hos raske personer med arvelig disposition for depression.

*Fordele*

Der er umiddelbart to fordele for forsøgsdeltagerne ved at deltage i forsøget:

- Af sikkerhedsmæssige årsager udtages veneblod ved forsøgets start til generel helbredsundersøgelse. Resultaterne af prøverne vil blive oplyst ved konsultation.
- Der gives oplysning om depression og der vil være mulighed for at diskutere forsøgspersonernes eventuelle bekymring for selv at udvikle sygdommen.

I øvrigt kan det ses som en fordel, at medvirke til at øge forståelsen af de psykiske og fysiologiske processer i hjernen, deres påvirkelighed af ssri og deres relation til genetisk og fænomenologisk sammensætning.

## **6.8. Bivirkningsregistrering**

Definition af hændelser/bivirkninger:

*Bivirkning:*

En bivirkning er alle skadelige og uønskede reaktioner på et lægemiddel uanset dosis.

*Uventede bivirkninger:*

En bivirkning, hvis karakter eller alvor ikke stemmer overens med produktoplysningerne.

*Hændelser*

En hændelse er enhver uønsket hændelse hos en forsøgsperson i et klinisk forsøg efter behandling med et lægemiddel, uden at der nødvendigvis er sammenhæng mellem denne behandling og den uønskede hændelse.

En hændelse kan således være ethvert ugunstigt eller utilsigtet tegn (inklusive et abnormt laboratoriefund) symptom eller sygdom, som midlertidigt er forbundet med anvendelsen af et lægemiddel, uanset om hændelsen anses for at have tilknytning til lægemidlet.

*Alvorlig bivirkning eller hændelse*

En hændelse eller bivirkning, som uanset dosis resulterer i død, er livstruende, medfører hospitalsindlæggelse eller forlængelse af hospitalsophold, resulterer i betydelig eller vedvarende invaliditet eller uarbejdsdygtighed.

## **6.9. Rapportering af hændelser og bivirkninger**

Alle uventede og alvorlige formodede bivirkninger rapporteres omgående til Lægemiddelstyrelsen af sponsor-investigator med kommentar om eventuelle konsekvenser for forsøget.

Sponsor-investigator indberetter alle oplysninger om uventede og alvorlige formodede bivirkninger, som er dødelige eller livstruende hurtigst muligt og senest 7 dage efter, at sponsor har fået kendskab til en sådan formodet bivirkning. Senest 8 dage efter indberetningen meddeler sponsor Lægemiddelstyrelsen alle relevante oplysninger om sponsor-investigators opfølgning af indberetningen. Alle andre uventede og alvorlige formodede bivirkninger indberettes til Lægemiddelstyrelsen senest 15 dage efter, at sponsor-investigator har fået kendskab til disse.

Sponsor-investigator vil foretage en årlig indberetning til Lægemiddelstyrelsen og Videnskabsetisk komite af alvorlige bivirkninger/hændelser.

Endvidere laves en slutrapport med samlet opgørelse af bivirkninger/hændelser (inklusive de alvorlige) som indsendes til Lægemiddelstyrelsen. Heraf vil i resumé-form fremgå oplysninger om antallet af behandlede forsøgsdeltagere, anvendte doser, doseringsvarighed, opnåede resultater samt observerede bivirkninger.

Anmeldelsesblanketter forefindes i Trial Master File.

## **6.10. Opfølgning ved hændelser eller bivirkninger**

Forsøgsdeltagerne vil blive fulgt af investigator eller afdelingen afhængig af hændelsens/bivirkningens karakter, så længe der vurderes behov for opfølgning.

## **6.11. Kriterier for forsøgs-/behandlingsstop**

Den enkelte forsøgsdeltager udgår af forsøget (dvs. seponering af forsøgsmedicinen/ophør af deltagelse i forsøget) såfremt:

- der opstår uacceptable bivirkninger eller alvorlige hændelser i forbindelse med behandlingen.

- der optræder tilstødende sygdom, der kompliceres af forsøgsmedicinen vurderet af

sponsor-investigator

- forsøgspersonen bliver gravid

- forsøgspersonen trækker sit samtykke tilbage

Forsøget stoppes som helhed såfremt, der tilkommer ny viden om området, der gør det uforsvarligt at fortsætte.

I øvrigt vil forsøget blive afsluttet når 80 evaluerbare forsøgspersoner har gennemført forsøget. Lægemiddelstyrelsen vil da straks blive informeret herom.

## **6.12. Forsøgspersoner der ønsker at udgå af AGENDA (drop-outs)**

Såfremt en forsøgsdeltager ønsker at træde ud af forsøget, vil denne blive kontaktet (telefonisk eller per brev) af sponsor-investigator med henblik på indsamling af oplysning om årsagen til ønsket om at træde ud af forsøget samt opfordring til at returnere ikke indtaget forsøgsmedicin.

Da det er hensigten at opnå i alt 80 evaluerbare forsøgsdeltagere, vil udgåede forsøgsdeltagere blive erstattet af nye forsøgsdeltagere.

## **6.13. Opfølgning i forsøgsperioden**

Forsøgsdeltagerne vil blive kontaktet telefonisk hver uge i forsøgsperioden med henblik på afklaring af evt. spørgsmål, bivirkninger mv. Forsøgsdeltagerne gives endvidere mulighed for at kontakte Ulla Knorr eller Lars V. Kessing ved akut behov eller ved behov i øvrigt i dag-tiden og instrueres i at kontakte Psykiatrisk Modtagelse, Rigshospitalet, ved akut behov i aften-nat perioden. I øvrigt vil compliance blive overvåget ved, at forsøgsdeltagerne anmodes om at medbringe al emballage vedr. forsøgsmedicinen på sidste undersøgelsesdag.

## **6.14. Effektmål, materialestørrelsesberegning og statistiske analyser.**

Effektmål vil blive sammenlignet for behandlings- og placebogruppen efter 4 ugers behandling med Cipralex®.

Primært effektmål defineres som forskellen på AUC–total (areal under responskurven fra indgift af CRH (kl. 15.00 ) til tidspunktet for sidste plasma-cortisolbestemmelse (kl. 18.00), ikke-baseline-korrigeret) for plasma-cortisol ved den kombinerede dexamethason corticotropin-releasing hormontest målt før og efter 4 ugers behandling med Cipralex® eller placebo. Det er muligt ved praktiske procedure at sikre blindingen. Bioanalytikeren som foretager målingerne vil ikke have kendskab til forsøgets detaljer og vil kun vide at prøverne er relateret til AGENDA.

Udfra resultater fra Modell et al´s højrisiko-studie [88] vurderes forsøgsdeltagerne i gennemsnit at have værdier svarende til højrisiko-probanderne i studiet:

Cortisol (nmol/L x min/L) AUC-total (mean ± SEM): 15064 ± 3947.

Klinisk betydende virkning af escitalopram behandling er, at værdierne reduceres til niveauet for værdierne for raske kontrolpersoner, hvilket i Modell´s studie er:

Cortisol (nmol/L x min/L) AUC-total (mean ± SEM): 7773 ± 1071.

Af økonomiske og etiske årsager er det fundet optimalt at inkludere i alt 80 forsøgsdeltagere med fuldt datasæt. Den relevante difference forsøget ønsker at detektere = 15064 – 7773 = 7291 og givet at SD = SEM x √14) = 3947 x 3,7 = 14768 opnås en styrke i forsøget på minimum 60 % (1 – β = 0,60) hvor β = risikoen for at overse en forskel på AUC-total.

Sekundære effektmål defineres som ændringer i score på kognitive tests og ændringer på neuroticisme-skalaen på EPQ.

Tertiære effektmål defineres som ændringer i det psykomotoriske tempo målt som gennemsnitlig aktivitet over tre døgn, ændringer i søvnmønsteret målt som ændringer i point på spørgsmål vedr. søvn målt med HAM-D foruden antal timers søvn og søvnkvalitet målt på VAS, samt ændringer i scores på HAM-D, BDI , NEO-PI-R, WHOQOL– BREF og Perceived Stress Scale, samt ændringer af GR-α mRNA ekspression, ændringer i plasmaproteinekspression (proteomics) og mmunologiske parametre.

Statistiske analyser foretages med hensyntagen til udgangsniveauet i kovariansanalyser (”analyses of covariance” (ANCOVA)) og med angivelser af 95% konfidensintervaller for effektmål. P<0.05 anses for statistisk signifikant.

Ved manglende værdier foretages såvel analyse af forsøgspersoner med komplette data som multipel imputation (MI) af manglende værdier med MI-analyze (SASS ver. 9.1) og i tilfælde af forskel på analyserne betragtes resultaterne fra MI proceduren som forsøgets resultat. Det tilstræbes at opnå så høj en gennemførselsprocent som muligt, da det er afgørende for validiteten af resultaterne.

## **6.15. Data-management.**

For hver forsøgsperson findes alle indsamlede data i en Case Report File, som opfylder Lægelovens § 13 om lægers pligt til at føre ordnede optegnelser og Bekendtgørelse nr. 846, §9. Hver Case Report File indeholder en afkrydsningsliste over indeholdte dokumenter. Med henblik på at opretholde blindingen vil svaret for se- escitalopram blive tilsendt CTU (att. Jørn Wetterlev, RH 33-44, Blegdamsvej 9, 2100 Kbh. Ø) og opbevaret i bankbox indtil forsøgets praktiske del er afsluttet og databehandlingen påbegyndes.

Forsøgspersonerne registreres ikke i Det Grønne System og fremdeles heller ikke i det Danske Psykiatriske Centrale Forskningsregister.

I forbindelse med databearbejdning dobbeltindtastes data med henblik på validering.

SponsorInvestigator er ansvarlig for data. Data vil blive opbevaret i et aflåst, brandsikkert arkivskab på Psykiatrisk Afdeling, Rigshospitalet i 10 år efter forsøgets afslutning.

## **6.16. Procedure for brydning af kode til randomisering (emergency-break).**

Af sikkerhedsmæssige hensyn kan randomiseringskoden brydes i løbet af forsøget, såfremt der opstår alvorlige bivirkninger, der kan relateres direkte til forsøgsmedicinen eller hvis der opstår alvorlige hændelser for forsøgsdeltageren. Det er Ulla Knorrs og i dennes forfald Lars. V. Kessings suveræne beslutning om koden skal brydes. Proceduren er, at Ulla Knorr eller Lars V. Kessing kontakter CTU og giver oplysning om løbenr., navn og CPR. nr. på den aktuelle forsøgsdeltager, som koden ønskes brudt for. Uden for almindelige åbningstider (se 6.5.) kontaktes CTU på mobil 27145172.

## **6.17. Uafhængig datamonitorerings og sikkerhedskomite.**

En uafhængig datamonitorerings og sikkerhedskomite er nedsat for yderligere at sikre forsøgspersonerne. Sikkerhedskomitéen kontaktes ved enhver alvorlig bivirkning eller hændelse under forsøget og tager stilling til om forsøget skal stoppes eller kan fortsætte. Der henvises til en nøjere udarbejdet skrivelse om komitéens arbejde.

## **6.18. Monitorering i henhold til reglerne om Good Clinical Practice (GCP).**

AGENDA vil blive gennemført efter denne forsøgsprotokol, ICH-GCP guideline, gældende myndighedskrav og lovgivning, når Lægemiddelstyrelsen, De Videnskabsetiske Komitéer for Københavns og Frederiksberg Kommuner og Datatilsynet har godkendt forsøgsprotokollen og GCP-enheden har gennemført initieringsbesøg. Københavns Universitets Hospitals GCP-enhed vil herudover monitorere forsøget i henhold til gældende regler og Sponsor-investigator tillader direkte adgang til kildedata/dokumenter ved monitorering, auditering og/eller inspektion fra henholdsvis en etisk komité, Lægemiddelstyrelsen eller fra andre landes sundhedsmyndigheder. Forsøget afsluttes når 80 forsøgsdeltagere, som har gennemført forsøget efter protokollen.

Sponsor-investigator har gennemgået e-learning kursus i GCP-regler.

# 7. Fremtidige undersøgelser

Med henblik på fremtidige genanalyser opbevares en blodprøve i Dansk Psykiatrisk Biobank.

Et vist antal af forsøgsdeltagerne vil blive spurgt, om de vil deltage i et parallelt forskningsforsøg vedr. PET-skanning af hjernen (selvstændig protokol vil blive fremsendt herfor).

# 8. Tidsplan

September 2005 påbegyndte det under pkt. 6.1. nævnte projekt, som forventes at kunne inkludere 500 patienter i løbet af 2 år. Af disse vil 40-50 % have et eller flere børn, som opfylder inklusionskriterierne. Foreløbigt er 159 patienter blevet undersøgt og tilsagn er opnået til at kontakte 67 arveligt disponerede forsøgsdeltagere. Med en deltagerandel på ca. 30-40 % kan således inkluderes ca. 80 forsøgsdeltagere. **Yderligere indkaldes søskende til patienterne med kendt depression som ovenfor beskrevet.**

Den forventede deltagerandel bygger på erfaringerne fra sammenlignelige undersøgelser foretaget på Psykiatrisk Klinik, Rigshospitalet, som har opnået deltagerprocenter på ca. 50 %. Herværende studium er muligvis mere krævende for forsøgsdeltagerne, hvorfor estimatet af deltagerprocenten er nedjusteret.

Ulla Knorr har været ansat fra 1.2. – 30.6.06 som klinisk assistent på Psykiatrisk Klinik, Rigshospitalet, har i perioden 1.7.06 – 31.1.09 ansættelse som forskningsassistent på Center for Pharmacogenomics, Farmakologisk Institut, Københavns Universitet og er den 1.6.2006 blevet indskrevet som ph.d.-studerende ved Det Sundhedsvidenskabelige Fakultet, Københavns Universitet.

Endelig koordinering og indkøring af forsøget berammes til……………………..… …..12 mdr.

Rekruttering af forsøgsdeltagere påbegyndes 1.2.2006 og løber over………….….……. 14 mdr.

Udfærdigelse af artikler samt ph.d.-afhandling berammes til……………………………….5 mdr.

Deltagelse i forskerkurser……………………………………………………………….….. 4 mdr.

Deltagelse i internationale kongresser……………………………………………………….1 mdr.

I alt………………………………………………………………………………………….36 mdr.

# 9. Rapportering

Resultaterne præsenteres på videnskabelige møder og kongresser og publiceres i form af mindst 3 publikationer svarende til de 3 grundhypoteser:

1. Association mellem polymorfier i serotonin transporteren og hhv. neuroticisme, kognitiv dysfunktion og dysregulation af HPA-aksen hos raske personer med arvelig disposition for depression.
2. Sammenhæng mellem hhv. neuroticisme, kognitiv dysfunktion og dysregulation af HPA-aksen hos forældre med depression og deres raske voksne **søskende eller** børn.
3. Effekt af behandling med escitalopram på hhv. neuroticisme, kognitiv dysfunktion og dysregulation af HPA-aksen hos raske personer med arvelig disposition for depression.

Data indhentet i forsøget, som ikke er rapporteret i de primære publikationer, kan indgå i publikationer eller efterfølgende projekter indenfor forskningsgruppen efter aftale med Ulla Knorr, der i så fald er medforfatter på publikationer.

The International Comittee of Medical Journal Editors retningslinier for medfatterskab vil blive respekteret [89].

Forsøget anmeldes på [www.clinicaltrials.gov](http://www.clinicaltrials.gov/).

# 10. Interessekonflikter

Ulla Knorr er ansat af Center for Pharmacogenomics, Københavns Universitet. Lars V. Kessing er ansat i et 5-årigt forskningsprofessorat ved København Universitet finansieret via Lundbeckfonden. Lundbeckfonden og H. Lundbeck A/S har ingen indflydelse på idé, protokol, analyser eller udfærdigelse eller publikation af undersøgelsen. H. Lundbeck A/S har adgang til data fra ekspressions-analyser.

# 11. Etiske overvejelser

Forsøget er ikke tidligere blevet gennemført og det vil kunne bidrage med ny viden om ætiologiske aspekter ved depression og vil således kunne give øget viden om mulighederne for forebyggelse og tidlig intervention ved depression.

I forsøget indgår kun myndige personer, som ikke er gravide eller ammende. Det er forbundet med et tidsforbrug på ca. 18 timer fordelt over 4 dage til interview og undersøgelse ved læge, besvarelse af spørgeskemaer, neuropsykologiske test, blodprøvetagning og neuroendokrinologisk stimulationstest. Herudover behandles forsøgsdeltagerne i 4 uger med antidepressiv medicin (escitalopram) eller placebo.

Medicinen gives i mindste anbefalede dosis, hvor der kun yderst sjældent ses alvorlige bivirkninger. Den alvorligste af disse skønnes at være serotonergt syndrom, hvilket som anført i afsnit 6.7. forekommer yderst sjældent og oftes når medicin gives i kombination [90]. Det er omdiskuteret, om behandling med escitalopram i sjældne tilfælde kan udløse suicidale impulser eller eufori hos patienter, som lider af depression [91-94]. Der foreligger ikke meddelelser om udløsning af serotonergt syndrom, suicidale impulser eller eufori hos raske personer, som er behandlet med escitalopram. Disse bivirkninger må forventes at forekomme yderst sjældent hos raske personer med arvelig disposition for depression. Øvrige bivirkninger forekommer, som beskrevet under punkt 6.7.og i deltagerinformationen. Eksklusionskriterierne der udelukker personer, der får medicin med kendt interaktion med Cipralex®, er valgt for at minimere risikoen for alvorlige bivirkninger.

Forsøgsdeltagerne vil blive kontaktet telefonisk ugentligt i forsøgsperioden med henblik på afklaring af evt. spørgsmål, bivirkninger mv. Forsøgsdeltagerne gives endvidere mulighed for, at kontakte Ulla Knorr eller Lars V. Kessing ved akut behov eller ved behov i øvrigt i dagtiden og instrueres i at kontakte Psykiatrisk Modtagelse, Rigshospitalet, ved akut behov i aften-nat perioden.

Forsøgsdeltagerne honoreres, jf. deltagerinformationen. Honoraret er fastsat under hensyntagen til de ulemper som forsøgsdeltagerne har i form af transporttid, undersøgelsestid, antal fremmøder, risiko for bivirkninger og tabt arbejdsfortjeneste. Honoraret kan justeres, hvis Komiteen bedømmer det. Forsøget udgår fra Psykiatrisk klinik, Rigshospitalet, i samarbejde med Københavns Universitet. H. Lundbeck A/S er ansøgt om støtte til honorering af forsøgsdeltagerne.

Nordmedica giver økonomisk støtte ved indkøb af CRH.

Fra Savværksejer Jeppe Juhl og Hustru Ovita Juhls Mindelegat er modtaget til gennemførelse af de neuropsykologiske undersøgelser.

I henhold til Lægemiddelloven § 88, stk. 1., ansøges Lægemiddelstyrelsen om tilladelse til forsøgets gennemførelse via www.lægemiddelstyrelsen.dk.

Forsøget vil efterleve Good Clincal Practice reglerne i henhold til direktiv 2001/20/EF [95] og monitoreres af Good Clincal Practice-enheden Gentofte Amtssygehus.

Forsøget er oprettet i Eudract. med nr. 2006-001750-28.

Forsøget er omfattet af Persondataloven af 1. juli 2000, §§ 7 og 8 og godkendt af Datatilsynet som privat forskningsforsøg efter anmeldelse via [www.datatilsynet.dk](http://www.datatilsynet.dk/).

Forsøget vurderes således med dets etiske aspekter at være i overensstemmelse med Helsinki-Deklarationen, World Medical Association, 2002 ([www.wma.net/e/policy/b3.htm](http://www.wma.net/e/policy/b3.htm)) og er godkendt af Videnskabsetisk Komité for København og Frederiksbergs kommuner.

# 12. Deltagerinformation

Dato

**Kære**

Vi vil spørge, om du vil deltage i det kliniske forsøg AGENDA, som udgår fra Affektiv Forskningsenhed, Psykiatrisk Klinik, Rigshospitalet.

**Hvordan har vi fundet frem til dit navn og adresse ?**

Den ene af dine forældre eller søskende har deltaget i et forskningsprojekt om depression og har i den forbindelse venligst givet tilladelse til, at vi måtte kontakte dig og spørge om du vil deltage i AGENDA. I alt har omkring 100 forældre eller søskende givet et sådant tilsagn.

**AGENDA** er en forkortelse for ”Associationer mellem genpolymorfier, endofænotyper for depression og antidepressiv behandling”.

I det følgende vil vi beskrive AGENDA og hvis du efter gennemlæsning har spørgsmål, er du velkommen til at kontakte os. Vores telefonnumre og e-mailadresser står på den sidste side. Du vil få honorering for deltagelse i forsøget og får dækket udgifter i form af tabt arbejdsfortjeneste og udgifter til transport.

**Baggrund for forsøget**

Med AGENDA ønsker vi at belyse sammenhængen mellem arveanlæg, hormoner, personlighedstræk, tænkning og behandling med antidepressiv medicin (Cipralex® - er en såkaldt ”lykkepille”). *Resultaterne* vil bidrage til forståelsen af, hvilke faktorer der medvirker til, at en person udvikler depression og vil belyse, hvordan disse faktorer påvirkes af behandling med medicin. Vi ved, at depression i en vis grad er arvelig, så derfor har vi valgt, at undersøge personer hvor depression forekommer i familien.

**Hvad indebærer forsøget for dig ?**

AGENDA afvikles over en måned og du skal møde 4 dage.

Den første dag gennemføres **A**, **B**, **C** og **D.** Få dage efter gøres **E** og **F**. Efter en måned gentages **B**, en mindre del af **C** og **E.** På den sidste dag gennemføres **E** og en afsluttende lægesamtale.

**A**. **Interview**

Ca. to timers varighed om fysisk og psykisk helbred.

**B**. **Spørgeskemaer**

Spørgsmål om social funktion, symptomer på depression og eventuelle medicinbivirkninger.

**C**. **Blodprøver**

Genanalyser, almindelig helbredsundersøgelse og til måling af eventuel Cipralex®. Blodprøverne til almindelig helbredsundersøgelse, inkl. evt. graviditetstest vil blive analyseret inden for et par dage og resten bliver opbevaret til senere analyse.

**D. Neuropsykologisk undersøgelse.**

Til denne del af undersøgelsen bedes du medbringe eventuelle læsebriller.

**E**. **Test af binyrefunktion**

Der gives overordnede stresshormoner i form af tablet dexamethason og i en blodåre gives Corticotropin-releasing hormon (CRH). Hormonstatus måles hvert kvarter inden for fire timer.

###### Bivirkninger for dexamethason er kortvarige og forbigående. Det drejer sig om rastløshed, søvnproblemer, psykisk påvirkning, højt blodsukker og forhøjet blodtryk. Ved CRH forekommer lettere bivirkningerne hos 20% og sjældent optræder generende rødmen og varmefornemmelse af ca. ½ times varighed.

**F**. **Medicinering**

Hver dag i 4 uger tages enten tablet Cipralex® 10 mg eller en uvirksom kalktablet

(placebo). Der bliver trækket lod om, hvilken behandling du vil få, men hverken du eller forsøgets stab vil være klar over, om du får Cipralex® eller placebo.

Cipralex® har udbredt anvendelse i behandlingen af depression, panikangst, social fobi eller generaliseret angst. Som al anden medicin kan Cipralex® give bivirkninger. Som regel er bivirkningerne milde og forsvinder efter få dages behandling. De hyppigst forekommende bivirkninger er kvalme, tilstoppet eller løbende næse, nedsat appetit, søvnproblemer, svimmelhed, diarré, forstoppelse, øget svedtendens, træthed, feber og så længe Cipralex® gives kan seksuallivet fungere mindre godt. I meget sjældne tilfælde kan ses mere alvorlige bivirkninger, som vi vil informere dig nærmere om. Efter forsøget trappes medicinen ned og du får ½ tablet i fem dage.

Vi vil tilbyde at ringe til dig i løbet af forsøget og høre hvordan det går. Hvis du er i tvivl om noget eller ønsker mere information, kan du i dagtiden kontakte enten Ulla Knorr eller Lars V. Kessing. Ved akut behov herudover kan Psykiatrisk Modtagelse Rigshospitalet kontaktes på tlf. 35456202. Såfremt du får behov for at tage anden medicin i løbet af behandlingsperioden skal Ulla Knorr eller Lars V. Kessing oplyses herom.

**For kvinder**

Det er væsentlig at du ikke bliver gravid i forsøgsperioden. Fosterskader forekommer ikke hyppigere under Cipralex® behandling end ved almindelige graviditeter, men en graviditet kan påvirke forsøgsresultaterne i betydelig grad. Hvis du ikke bruger sikker prævention vil vi gerne foretage en graviditetstest ved forsøgets start og slutning.

**Yderligere undersøgelser**

Der kan også blive mulighed for at deltage i et parallelt forskningsforsøg vedr. hjerneskanning.

**Frivillighed**

Det er frivilligt, om du vil deltage i forsøget og såfremt du ønsker at deltage, står det dig frit for, til hver en tid at træde ud igen, hvis du skulle ønske det. Hvis der er dele af undersøgelsesprogrammet du ikke skulle have lyst til at deltage i, kan du deltage i de øvrige dele.

Før undersøgelserne går i gang, får du detaljeret mundtlig information om forsøget. Du er velkommen til at tage en ledsager med, hvis du ønsker det. Du får også Den Centrale Videnskabsetiske Komités folder med oplysninger om aktindsigt, klageadgang m.m.

**Godkendelser**

Forsøget er godkendt af Den Videnskabsetiske Komité for Københavns og Frederiksberg kommuner; journalnr. KF 02-307413, H-KF-307413, Lægemiddelstyrelsen og Datatilsynet. Blodprøvetagning til Den Psykiatriske Biobank er godkendt af Den Videnskabsetiske Komité for Københavns og Frederiksberg kommuner; journalnr. KF 01-024/01.

**Hvordan sikres anonymitet ?**

De fortrolige oplysninger, som fremkommer i forbindelse med forsøget er omfattet af tavshedspligt. Forsøgsresultaterne fra forsøget vil blive publiceret i videnskabelige tidsskrifter i anonymiseret form. Når forsøget afsluttes vil alle personrelaterede oplysninger blive slettet.

**Hvordan bliver du honoreret ?**

Deltagelse i forsøget honoreres med 1000 kr. pr. undersøgelsesdag. Du vil herudover få 1000 kr. pr. undersøgelsesdag for tabt arbejdsfortjeneste. Dine transportudgifter dækkes svarende til udgiften ved offentlig transport alt efter, hvor langt du bor fra Rigshospitalet. Evt. deltagelse i tre skanninger vil blive honoreret med 500 kr. per skanning. Honoraret er B-skattepligtigt. Det maximale beløb kan blive 9.500 kr. plus transport. Forskellige forhold vil kunne forhindre, at du kan gennemføre hele undersøgelsesprogrammet og honoreringen vil da kunne blive mindre I gennemsnit skønnes honoreringen beløbe sig på 6500 kr. Dersom du deltager i den indledende første-dagsundersøgelse og det viser sig at vi ikke kan risikere at give dig forsøgsmedicinen, vil du få honoraret for første dag, dvs.2000 kr., samt transportgodtgørelse.

**Økonomisk støtte til forsøget**

Forsøget udgår fra Psykiatrisk klinik, Rigshospitalet, i samarbejde med Københavns Universitet.

H. Lundbeck A/S støtter honorering af forsøgsdeltagere.

Vi håber, at du vil deltage og at du kontakter os telefonisk, skriftligt eller pr. Email for yderligere oplysninger. Hvis vi ikke hører fra dig, tillader vi os at kontakte dig igen indenfor en måned enten telefonisk eller skriftligt.

Venlig hilsen

Ulla Knorr, 1. reservelæge, klinisk assistent og Lars V. Kessing, professor, overlæge, dr.med.,

Psykiatrisk Klinik, Rigshospitalet,

Borgmester Jensens Allé 55,

2100 København Ø.

Tlf. 35 45 62 35 eller 35 45 61 77

Email: [ulla.knorr@rh.regionh.dk](mailto:ulla.knorr@rh.hosp.dk) og [lars.kessing@rh.dk](mailto:lars.kessing@rh.dk)

**Samtykkeerklæring og Fuldmagtserklæring**

**Vedrørende**

**Forskningsforsøget AGENDA**

Jeg bekræfter hermed, at jeg – efter at være blevet orienteret om ovennævnte forsøg, både mundtligt og skriftligt – gerne vil deltage i det beskrevne forsøg, og at jeg har fået et underskrevet eksemplar af deltagerinformationen, samtykkeerklæring og fuldmagtserklæring.

Jeg er blevet orienteret om, at det er frivilligt at deltage, og at jeg kan trække mit tilsagn om deltagelse tilbage på et hvilket som helst tidspunkt uden, at det får konsekvenser for min eventuel fremtidige behandling og kontroller på afdelingen.

Jeg er klar over, at der vil blive taget blodprøver, som vil blive analyseret ved en senere lejlighed.

Jeg er klar over, at forsøget kan blive afsluttet på et hvilket som helst tidspunkt uden mit samtykke i det tilfælde, at der skønnes af foreligge en sikkerhedsrisiko eller hvis myndighederne kræver det.

Undertegnede giver hermed fuldmagt til, at forsøgsoptegnelser kan blive set af repræsentanter fra Københavns Universitetshospitals Good Clinical Practice enhed og dertil tilknyttede personer, danske og udenlandske lægemiddelmyndigheder samt Den Videnskabsetiske Komité med det formål at kontrollere oplysningernes rigtighed i forbindelse med monitorering, audit eller inspektion. Fuldmagten er gældende under forsøget og i indtil 10 år efter forsøgets afslutning. Fuldmagten kan til enhver tid tilbagekaldes. Jeg er blevet orienteret om, at alle data behandles fortroligt.

Denne tilladelse gælder kun for oplysninger, som har med dette forsøg at gøre og tilladelsen kan trækkes tilbage på et hvilket som helst tidspunkt.

________ ______________________ _____________________________

Dato Forsøgsdeltagerens navn Underskrift

(Blokbogstaver)

________ _ Ulla Knorr ___ ____________________________

Dato Informerende læges navn Underskrift

Jeg vil eventuelt gerne kontaktes senere med henblik på opfølgende spørgsmål eller forespørgsel om deltagelse i en opfølgende undersøgelse.

Ja ___ Nej __

# 13. Referencer

**Bilag 1 Agenda-blodprøver**

| **Prøve** | **Glas, ml** | **Behandling** | **Analysested** | **Antal** |
| --- | --- | --- | --- | --- |
| P-Kalium-ion, stofk  P-Natrium-ion, stofk.  B-Hæmoglobin (Fe), stofk.  B–Leucocytter, antal,  B–Leucocytter, gruppe  B- Thrombocytter, antalk.  P-Glucose,stofk.,  P-Lactat-dehydrogenase, enzk.,  P-Alanin-aminotransferase, enzk.,  P-Aspartat- aminotransferase, enzk.,  P-Basisk phosphatase, enzk.,  P-Bilirubin, stofk., (total),  P-C-reaktivt protein; massek.  P-Creatinin, stofk.,  P-Thyreotropin, arb. stofk. (=TSH),  P-cortisol  P-acth  P-Cholesteroler, stofk.  P-Cholesteroler, HDL, stofk.  P-Cholesteroler, LDL, stofk  P-Triglycerid, stofk.  s-HCG (kun kvinder) | Lilla, gråt og  lysegrønt | Tages i blodbanken  Køleskab | Klinisk biokemisk, almindelig drift | 80 x 2=160 |
| Genanalyser | Lilla 10ml | Tages i blodbanken – 80 grader.  Flyttes ca. 5 ad gangen til Neuropsykiatrisk lab. | Psykiatrisk biobank | 240 |
| **RNA** | 4 Paxgene tube fra BD, a  (2,5 ml)  Bestilles på 80885945 100 ad gangen | Stuetemperatur  Skal vendes 10 gange  Kan holde sig i 6-12 måneder på frys –20 - -80. | Center for Pharmacogenomics | 320 |
| **Immunologiske parametre (cytokinprofiler)** | **a)**  **Cytokiner:**  **2 EDTA lilla 6 ml**  **b)**  **Flowprofil:**  **KLM-155**  **(lyseblå 2,7)**  **EDTA** | **a)**  **Køles straks og kølecentrifugeres indenfor en time og fryses –80 g.**  **b) Stuetemperatur.** | **a) Blodbanken**  **b) Blodbanken** | 2 x 80 x 2**=320**  1x2x80**=160** |
| **Fuldblodsstimulation** | **Mørkegrønt 10 ml heparin** |  | **Blodbanken** | 160 |
| **Tromboelastografi** | **Blå citrat, 2,7 ml** |  | **Blodbanken** | 160 |
| **Proteomics** | **1 plasmaprøve**  **(EDTA )**  **(lilla 6 ml)**  **(rød 10 ml)** | **Tidspunkt noteres på glasset. Tages på knust is,og vendes EDTA glasset forsigtigt 10 gange. Kølecentrifugeres.**  **afpipeteres.** | **– 80 graders fryser** | 2x80x2 = **320** |
| *s-escitalopram | KLM-104  (rød 10 ml) | Almindelig drift | Lab Risskov via klinisk biokemisk på hvide sedler | 80 |
| p-cortisol | KLM-135 (lysegrøn 3ml) | Kan stå i fire timer ved stuetemperatur. Fryses til – 80 grader efter centrifugering. | Klinisk biokemisk | 1 + 16 x 2 x 80  = 2561 |
| p-ACTH + reserve | EDTA glas  KLM-115  (2 x lilla 3 ml) | Sættes ved stuetemp i 15 min. Kølecentrifugeres inden for 30 min. afpippeteres og fryses til –80 grader straks efter centrifugeringen i plastik glas. | Klinisk biokemisk, immulite,  fryses til – 80 efter aftale med Torben Pedersen | 16 x 2 x 80  = 2560 x 2 |
| Spytcortisol  (validering og hjemme) | Sarstedt salivette kit, ca. 2.40 kr pr. stk. | Kølecentrifugeres og fryses til –80 grader. Hjemmeprøver fryses i forsøgspersons egen fryser og medtages i kuvert til behandling samme dag som DEX-CRH. | Klinisk biokemisk | 1-2 + (16x2) + (8x2)  x 80 = 3842 |

*s-escitalopram : På almindelig rekvisitionsseddel skrives ”prøver til andre afdelinger” og en gul seddel udfyldes med oplysning om escitalopram. Sendes til klinisk biokemisk som sender prøven videre.

**Glas og øvrige remedier leveres fra Klinisk Biokemisk 3011 fraset Paxgene som bestilles separat.

******* Proteomics, immunologiske prøver og RNA mærkes med mærkater fra Klinisk Biokemisk; Agenda, A / B (for før/efter)

Forsøgsdeltageren følges af UK til afsnit 2034. På telefon 5-3473 adviseres bioanalytikerne Marie Bruun eller Janne Amstrup med oplysning om Agenda-nr.

**Bilag 2. Mærkning af prøver af stresshormoner**

| **AGENDA Fryser**  **Mærkning af prøveglas**  **(115 stk)** | **Forsøgsnr**  **2202** | **Deltagernr.**  **001-150** | **Glas-id.**  **001-999** |
| --- | --- | --- | --- |
| **Spyt cortisol (51)** |  |  |  |
| Baseline spyt cortisol (1) |  |  | 001 |
| Spyt cortisol hjemme - 1 (9) |  |  | 002 - 010 |
| Spyt cortisol under DEX-CRH-1  (16) |  |  | 011 - 026 |
| Spyt cortisol hjemme – 2 (9) |  |  | 027 - 035 |
| Spyt cortisol under DEX-CRH -2  (16) |  |  | 036 - 051 |
| **Plasma cortisol (32)** |  |  |  |
| DEX-CRH - 1  plasma cortisol (16) |  |  | 052 - 067 |
| DEX-CRH -2  plasma cortisol (16) |  |  | 068 - 083 |
| **Plasma ACTH (64)** |  |  |  |
| DEX-CRH – 1,  plasma ACTH (16) |  |  | 083 - 098 |
| DEX-CRH – 2,  plasma ACTH (16) |  |  | 099 - 114 |
| **Gemmeglas – 1 ( 16 )** |  |  | 115 - 130 |
| **Gemmeglas – 2 ( 16 )** |  |  | 131 - 146 |
| **Extra** |  |  | 147 - 170 |
| **Til spytrør, 200 stk med tal** |  |  | 0-9 |

**Bilag 3.**

KOMBINERET DEXAMETHASON- CORTICOTROPIN RELEASING HORMONE TEST (DEX-CRH) [96]

## Teoretisk baggrund

Testen udføres som led i diagnostisk udredning af depressive patienter. Den kan anvendes til prædiktion af respons på medicinsk antidepressiv behandling.

I forskningssammenhæng undersøges om DEX-CRH-responset er en endofænotype for depression.

## Kontraindikation og forsigtighedsregler

Stimulationstesten foregår under anafylaksi beredskab (Hjertebræt, Rubens ballon, inj. adrenalin 1 mg/ml, solucortef, antihistamin, NaCl ).

Der er ingen forsigtighedsregler eller kontraindiaktioner for CRH.

For dexamethason kan opstå beskedne bivirkninger i form af rastløshed og søvnbesvær døgnet efter.

## Forberedelser

- Forsøgspersonen skal have sovet om natten op til testdagen.
- Forsøgspersonen må ikke have skiftet tidszone inden for de seneste døgn.
- På testdagen skal forsøgspersonen skal have spist frokost kl. ca. 12.
- Kvindelige forsøgspersoner bør være i den follikulære fase, dvs. fra dag 1-13, efter 1. dag i seneste menstruation.
- Forsøgspersonen må ikke være i behandling med medikamenter, der har interaktion med corticosteroider, særligt ikke hormonelle antikontraceptiva eller corticosteroider (både systemiske, lokal steroid spray, øjendråber, steroid creme og blokader indeholdende steroid).
- Forsøget skal foregå i rolige omgivelser med forsøgspersonen liggende.
- Kl. 23.00 aftenen før CRH-testen gives 1,5 mg dexamethason (Decadron®) som tabletter.

## Procedure

- Kl. 13.30 møder forsøgspersonen.
- Venflon anlægges i stor antecubital vene og patienten hviler i rygleje i et roligt og veltempereret lokale. Forsøgspersonen må ikke bøje armen under forsøget.
- Prøvetagning påbegyndes kl. 14.00 og der tages prøver hvert 15. minut frem til kl. 18.00.

10 ml blod udtages hver gang, fordeles med i KLM-135 (lysegrøn 3ml) og isafkølet

KLM-115 (lilla 3 ml), der vendes forsigtigt 5-6 gange. Resten overføres til tørglas. Prøverne kølecentrifugeres, afpipetteres og så snart plasma er separeret fra blodcellerne fryses det ved –80 °C.

- Kl. 15.00 gives *100 μg humant CRH (Cortirel®)* opløst i 2 ml solvens som bolus over ½-1 min.
- Der skylles efter med 2-4 ml 0.9% NaCl med Heparin 100IE/ml efter hver prøvetagning.
- Prøver og blodprøvesedler mærkes med stregkoder.

**Utensilier**

- Venflon (grøn) og tre-vejshane.
- 16 stk. 15 ml sprøjter til blodprøvetagning.
- 16 stk. tørglas (KLM 105) (serum) mærket med stregkoder.
- 16 EDTA glas (KLM115) til ACTH (plasma) mærket stregkoder.
- Ampul med 100 μg humant CRH som tørstof. Solvens: Sterilt saltvand.
- Vandbad med is til ACTH glas.
- 0.9 % NaCl med heparin (100 IE/ml).
- Kølecentrifuge 4 °C
- Pipetter
- Glas mærket med stregkoder
- Fryser -80°C.

**14. september 1999**

**PRODUKTRESUMÉ**

**for**

**Decadron, tabletter**

0. D.sp. nr.

1787

1. Specialitetens navn

Decadron

2. Deklaration

Dexamethason 0,5 mg.

3. Lægemiddelform

Tabletter.

**4. KLINISKE OPLYSNINGER**

4.1 Indikationer

Tilstand og sygdomme, ved hvilke glukokortikoider er indiceret.

4.2 Dosering

*Generelle rekommandationer for oral administration*

Dosisbehov er varierende og skal fastsættes individuelt i forhold til sygdommens svær­hedsgrad og patientens respons. Sædvanlig initialdosis varierer fra 0,50 til 15 mg dagligt afhængig af sygdommen der behandles for (for spædbørn og børn vil det sædvan­ligvis være nødvendigt at reducere den anbefalede dosis, men dosis bør fastsættes efter hvor alvorlig tilstanden er frem for efter alder og kropsvægt).

Kortikosteroidbehandling erstatter ikke, men supplerer konventionel behandling, som bør institueres som indikeret.

Dosis bør reduceres eller behandlingen seponeres gradvist efter administration i mere end få dage.

Til akutte tilstande hvor prompte lindring er nødvendig, er det tilladt og kan være påkrævet at anvende store doser i kort tid. Når symptomerne er tilstrækkeligt under kontrol bør dosis holdes på det mindst mulige niveau der opretholder tilstrækkelig symptomlindring uden stor hormonal påvirkning.

Ved kroniske tilstande kan der opstå spontan remission. Hvis dette opstår skal behandling med kortikosteroider seponeres gradvist.

Rutine laboratorieundersøgelser som urinanalyse, 2-timers postprandialt blodsuk­ker, måling af blodtryk og kropsvægt samt røntgen af brystet bør foretages regelmæssigt ved længerevarende behandling. Periodisk måling af serum kalium tilrådes hvis der anvendes store doser.

Patienter kan overføres til Decadron fra alle andre glukokortikoider hvis dosis justeres korrekt.

Følgende milligramsækvivalenter gør det nemmere at skifte til Decadron fra andre glukokortikoider:

| DECADRON | 0,75 mg |
| --- | --- |
| Methylprednisolon og Triamcinolon | 4 mg |
| Prednisolon and Prednison | 5 mg |
| Hydrokortison | 20 mg |
| Kortison | 25 mg |

Dexamethason svarer ca. milligram for milligram til betamethason, 4-6 gange mere potent end methylprednisolon og triamcinolon, 6-8 gange mere potent end prednison og prednisolon, 25-30 gange mere potent end hydro­kortison og ca. 35 gange mere potent end kortison. Ved ækvipotente antiinflammatoriske doser mangler dexamethason næsten fuldstændig hydrokortisons og tæt relaterede hydrokortisonderivaters natriumbesparende egenskaber.

*Specifikke dosisrekommandationer*

Ved kronisk, sædvanligvis ikke-dødelig sygdom, inklusiv endokrinologiske og kroniske reumatiske lidelser, ødematøse tilstande, respiratoriske og gastrointestinale sygdomme, visse dermatologiske sygdomme og hæmatolo­giske tilstande, skal der startes med en lav dosis (0,5-1 mg dagligt) og dosis gradvist øges til den mindste dosis, der giver den ønskede symptom­lindring.

Dosis kan administreres 2, 3 eller 4 gange dagligt.

I kongenital adrenal hyperplasi, er den sædvanlige daglige dosis 0,5-1,5 mg.

Ved akut, ikke-dødelig sygdom, inklusiv allergiske tilstande, oftalmolo­giske sygdomme, akutte og subakutte reumatiske tilstande, varierer dosis mellem 2 og 3 mg dagligt; en højere dosis kan imidlertid være nødvendig hos nogle patienter. Eftersom udviklingen af disse tilstande er selvbegræn­sende, er længere tids vedligeholdelsesbehandling som regel ikke nødven­dig.

Antiemetisk profylakse under emetogen kemoterapi: I kliniske studier blev 8-20 mg dexamethason givet intravenøst over 5-15 minutter umiddelbart inden kemoterapi efterfulgt af 4 mg dexamethason oralt hver 4. til 6. time eller med 8 mg oralt hver 8. time og gradvist aftagende i enten styrke eller administrationsfrekvens over 2 - 3 dage. Generelt bør den totale behand­lingstid med denne indikation ikke overskride 5 dage efter kemoterapi. Alternativt blev dexamethason til injektion givet intravenøst i stedet for en oral formulering i forskellige skemaer (se *Kliniske undersøgelser* med henblik på yderligere information om dosis og administration).

Brug med andre antiemetiske midler: Nogle patienter har modtaget dexa­methason samtidig med ondansetron for at opnå forstærket effekt af anti­emetisk profylakse under cisplatin eller non-cisplatin emetogen kemoterapi. Dexamethasondosis anvendt ved samtidig behandling svarede til dosis ved enkeltbehandling (se *Kliniske undersøgelser* med henblik på yderligere information om dosis og administration).

Samtidig administrering af dexamethason og metoclopramide har også vist forstærket effekt ved antiemetisk profylakse under emetogen kemoterapi.

I akutte, selvbegrænsende allergiske tilfælde eller akut forværring af kronisk allergi (f.eks. akut allergisk rinitis, akutte anfald af sæsonbetonet astmatisk bronkitis, urticaria medikamentosus og kontaktdermatitis) foreslås følgende doseringsskema, der kombinerer parenteral og oral behandling:

|  |  | Total daglig dosis |
| --- | --- | --- |
| 1. dag | 1 eller 2 ml. (4 eller 8 mg) Decadron injektionsvæske intramuskulært | 4 eller 8 mg. |
| 2. dag | 2 tabletter Decadron á 0,5 mg 2 gange dagligt. | 4 tabletter |
| 3. dag | 2 tabletter Decadron á 0,5 mg 2 gange dagligt. | 4 tabletter |
| 4. dag | 1 tablet Decadron á 0,5 mg 2 gange dagligt. | 2 tabletter |
| 5. dag | 1 tablet Decadron á 0,5 mg 2 gange dagligt. | 2 tabletter |
| 6. dag | 1 tablet Decadron á 0,5 mg 1 gang dagligt. | 1 tabletter |
| 7. dag | 1 tablet Decadron á 0,5 mg 1 gang dagligt. | 1 tabletter |
| 8. dag | Opfølgningsbesøg |  |

Ved kronisk, potentielt dødelig sygdom som systemisk lupus erythematose, pemphigus og symptomatisk sarcoidose er den anbefalede initiale dosis 2-4,5 mg dagligt; nogle patienter kan have behov for større doser.

Når sygdommen er akut og livstruende (f.eks. akut reumatisk carditis, systemisk lupus erythematosekrise, alvorlige allergiske reaktioner, pemphi­gus og neoplastiske sygdomme) er den initiale dosis mellem 4 og 10 mg dagligt, administreret i mindst 4 adskilte doser.

Epinephrin er førstevalg ved alvorlige allergiske reaktioner. Decadron kan anvendes enten samtidig eller som supplement til behandlingen.

Ved cerebralt ødem, når vedligeholdelsesbehandling er påkrævet, til palliativ behandling af patienter med recidiverende eller inoperable hjerne­tumorer, kan en dosis på 2 mg 2-3 gange dagligt være effektiv. Der bør anvendes den mindste dosis nødvendig for at kontrollere cerebralt ødem.

Ved adrenogent syndrom kan daglige doser på 0,5-1,5 mg holde børn i remission og forhindre tilbagevenden af abnorm udskillelse af 17-keto­steroider.

Som massiv behandling ved særlige sygdomme, f.eks. akut leukæmi, det neprotiske syndrom og pemphigus, anbefales 10-15 mg dagligt. Patienter der modtager så høj en dosis bør følges tæt med henblik på alvorlige reaktioner.

*Dexamethson suppresionstest*

Tests for Cushing’s syndrom: Giv 1,0 mg Decadron oralt kl. 23. Tag blod­prøver med henblik på plasma cortisolværdier kl. 8 den efterfølgende morgen. For at opnå større nøjagtighed, giv 0,5 mg Decadron oralt hver 6. time i 48 timer. Der foretages 24-timers urinopsamling med henblik på måling af 17-hydroxykortikosteroidudskillelse.

*Test med henblik på at skelne mellem Cushing’s syndrom pga hypofyse ACTH overskud og Cushing’s syndrom pga andre årsager*: Giv 2,0 mg Decadron oralt hver 6. time i 48 timer. Der foretages 24-timers urinopsam­ling med henblik på måling af 17-hydroxykortikosteroidudskillelse.

4.3 Kontraindikationer

Systemiske svampeinfektioner (se 4.4 Særlige advarsler og forsigtighedsregler vedrørende brugen - amphotericin B).

Overfølsomhed over for et eller flere af produktets indholdsstoffer.

Administrering af levende virusvacciner (se 4.4 Særlige advarsler og forsigtighedsregler vedrørende brugen).

4.4. Særlige advarsler og forsigtighedsregler vedrørende brugen

Man bør anvende lavest mulig dosering af kortikosteroider til at behandle sygdommen og når reduktion af dosis er mulig, bør reduktionen ske gradvist.

Kortikosteroider kan forværre systemiske svampeinfektioner og bør derfor ikke anvendes hvis sådanne infektioner er tilstede med mindre det er nødvendigt med henblik på at kontrollere medicinreaktion pga. amphotericin B. Derudover har der været rapporteret tilfælde hvor samtidig brug af amphotericin B og hydrokortison blev efterfulgt af hjerteforstørrelse og kongestivt hjertesvigt.

Ifølge litteraturrapporter er der en åbenlys sammenhæng mellem brugen af kortikosteroider og venstresidig ventrikel fri væg-ruptur efter nylig myokardiel infarkt. Behandling med kortikosteroider bør derfor anvendes med stor forsigtighed hos disse patienter.

Gennemsnitlige og store doser af kortison eller hydrokortison kan forårsage stigning i blodtrykket, salt- og vandretention og forhøjet udskillelse af kalium. Det er mindre sandsynligt at dette forekommer med syntetiske derivater bortset fra når anvendt i store doser. Saltfattig diæt og kaliumtilskud kan være nødvendigt. Alle kortikosteroider forøger udskillelsen af kalcium.

Lægemiddelinduceret nedsat sekundær adrenocortical funktion kan være forårsaget af for hurtigt seponering af kortikosteroider og kan mindskes ved gradvis reduktion af dosis. Denne type relativ nedsat funktion kan persistere måneder efter seponering af behandling. Kortikosteroidbehandling bør derfor reinstitueres eller dosis øges hvis der opstår en stress-situation i denne periode. Da mineralkortikoid sekretion kan være nedsat bør der samtidig administreres salt og/eller mineralkortikoid.

Efter længerevarende behandling kan seponering af kortikosteroider forårsage withdrawalsyndrom, der inkluderer feber, myalgi, artralgi og almen utilpashed. Dette kan også opstå hos patienter uden at der er tegn på nedsat adrenalfunktion.

Administration af levende vacciner er kontraindiceret hos patienter der modtager immunsuppresive doser af kortikosteroider. Hvis der administreres inaktiverede virale eller bakterielle vacciner der modtager immunsuppressive doser af kortikosteroider, opnås den forventede serum antistof respons muligvis ikke. Immuniseringsprocedurer kan dog foretages hos patienter der modtager kortikosteroider som erstatningsbehandling, f.eks. for Addisons sygdom.

Brugen af Decadron ved aktiv tuberkulose bør begrænses til tilfælde af fulminant eller dissemineret tuberkulose hvor kortikosteroider anvendes til at administreres sygdommen i forbindelse med et passende antituberkuloseregi­me. Hvis kortikosteroider er indiceret til patienter med latent tuberkulose eller tuberkulin reaktivitet er nøje observation nødvendig da sygdommen kan reaktiveres. Ved længerevarende kortikosteroidbehandling bør disse patienter modtage kemoprofylakse.

Steroider bør bruges med forsigtighed i tilfælde af nonspecifik ulcerativ colitis hvis der kunne være perforation, abscesser eller anden pyogen infektion, divertikulitis, intestinal anastomose, aktivt eller latent peptisk ulcus, nedsat nyrefunktion, hypertension, osteoporose og myasthenia gravis. Tegn på peritoneal irritation efter gastrointestinal perforation hos patient der modtager store doser kortikosteroider kan være minimal eller ikke-eksisterende. Fedtembolisme er rapporteret som mulig komplikation ved hyperkortisonisme.

Patienter med hypothyroidisme og med cirrhosis har forstærket effekt af kortikosteroider. Steroider kan forstærke eller mindske antallet og motiliteten af spermatozo hos nogle patienter.

Kortikosteroider kan maskere visse tegn på infektion og nye infektioner kan opstå under brugen.

Ved cerebral malaria kan brugen af kortikosteroider være associeret med forlængelse af koma og en højere incidens af pneumoni og gastrointestinal blødning.

Kortikosteroider kan aktivere latent amebiasius eller strongyloidiasis eller forværre aktiv sygdom. Det anbefales derfor at latent eller aktive amebiasis og strongyloidiasis udelukkes inden påbegyndelse af kortikosteroidbehandling hos patienter med risiko for eller symptomer der kunne tyde på disse sygdomme.

Længere tids brug af kortikosteroider kan give posterior subkapsulær katarakt, glaukom med mulig skade på de optiske nerver and kan øge risikoen for sekundære okulære infektioner pga. svamp eller virus.

Kortikosteroider bør anvendes med forsigtighed hos patienter med okulær herpes simplex pga. mulig korneaperforation.

Spædbørns og børns vækst og udvikling bør nøje følges ved længerevarende kortikosteroidbehandling.

Patienter som modtager immunsuppressiv medicin har større risiko for infektioner end raske. For eksempel kan skoldkopper og mæslinger have alvorligere, måske endda fatale, forløb hos ikke-immune børn eller voksne på koritkosteroider. Hos børn og voksne, som ikke har haft disse sygdomme, bør der tages særlige forholdsregler for at undgå smittefare. Risikoen for at udvikle disseminerede infektioner varierer fra individ til individ og kan hænge sammen med dosis, regime og behandlingslængde med kortikosteroider samt den underliggende sygdom. Patienter der har været udsat for smitte bør rådes til at søge prompte lægehjælp. Hvis patienten har været udsat for mæslinger kan profylaktisk behandling intramuskulært poolet immunglobulin (IG) være indiceret. Hvis patienten har været udsat for skoldkopper kan profylaktisk behandling med varicella zoster immunglobulin (VZIG) være indiceret. Hvis der udvikles skoldkopper bør behandling med antiviralt middel overvejes.

4.5 Interaktioner

Acetylsalicylsyre i kombination med kortikosteroider bør anvendes med forsigtighed hos patienter med hypoprothrombinæmi.

Phenytoin (diphenylhydantoin), phenobarbital, efedrin og rifampin kan øge den metaboliske clearance af kortikosteroider, hvilket resulterer i lavere blodni­veauer og mindre fysiologisk aktivitet hvilket kræver justering af kortiko­steroiddosis. Disse interaktioner kan interferere med dexamethason suppres­sionstest, som skal tolkes med forsigtighed ved administration af disse lægemidler.

Der er rapporteret falsk negative resultater i dexamethason suppressionstesten hos patienter i behandling med indomethacin.

Prothrombintiden bør kontrolleres hyppigt hos patienter i behandling med kortikosteroider og coumarin antikoagulanter samtidig da er set eksempler på, at kortikosteroider har ændret responsen til disse antikoagulanter. Under­søgelser har vist at effekten ved samtidig behandling med kortikosteroider sædvanligvis er hæmning af responsen til coumariner - der har dog været modstridende rapporter om potensering, der ikke er underbygget af under­søgelser.

Når kortikosteroider administreres samtidig med kaliumdepleterende diuretika bør patienten observeres omhyggeligt med henblik på udvikling af hypokaliæ­mi.

Kortikosteroider kan påvirke nitroblue tetrazolium testen for bakteriel infektion og forårsage falsk-negative svar.

4.6 Graviditet og amning

*Graviditet:*

Da der ikke er udført humane reproduktionsundersøgelser med kortikosteroider kræver brugen af disse lægemidler hos gravide eller potentielt gravide kvinder at de forventede fordele nøje afvejes i forhold til mulige risici for mor og foster. Spædbørn født af mødre som har modtaget større doser kortikosteroider i graviditeten bør nøje observeres med henblik på tegn på hypoadrenalisme.

*Amning:*

Kortikosteroider udskilles i brystmælk og kan hæmme væksten, interferere med endogen kortikosteroidproduktion eller forårsage andre uønskede virkninger hos det diende spædbarn. Mødre der tager farmakologiske doser af kortikosteroider bør frarådes at amme.

4.7 Trafikfarlighed

Der er bivirkninger forbundet med dette produkt, der kan påvirke visse patienters evne til at føre motorkøretøj og betjene maskiner (se 4.8 Bi­virkninger).

4.8 Bivirkninger

*Væske- og elektrolytforstyrrelser*:

Natriumretention.

Væskeretention.

Kongestivt hjertesvigt hos disponerede patienter.

Kaliumtab.

Hypokaliæmisk alkalosis.

Hypertension.

*Muskelskeletal:*

Muskelsvækkelse.

Steroid myopati.

Tab af muskelmasse.

Osteoporose.

Vertebrale kompressionsfrakturer.

Aseptisk nekrose af femoral- og humeralhovedet.

Patologisk fraktur af langknogler.

Seneruptur.

*Gastrointestinale:*

Peptisk ulcus med mulig perforation og blødning.

Hæmorrhagisk perforation af tynd- og tyktarm, specielt hos patienter med inflammatorisk tarmsygdom.

Pankreatitis.

Abdominal distension.

Ulcerativ esophagitis.

*Dermatologisk*

Dårlig sårheling.

Tynd, sart hud.

Petechiae og ecchymoser.

Erythem.

Øget svedtendens.

Kan hæmme hudtestreaktioner.

Andre kuntanreaktioner, som allergisk dermatitis, urticaria, angioneurotisk ødem.

*Neurologisk:*

Kramper.

Øget intrakranielt tryk med papilødem (pseudotumor cerebri) sædvanligvis efter behandling.

Vertigo.

Hovedpine.

Psykiske forstyrrelser.

*Endokrinologisk:*

Menstruationsforstyrrelser.

Udvikling af cushingoid tilstand.

Væksthæmning hos børn.

Manglende sekundær adrenokortikal og hypofyse respons, specielt ved stress, som ved traumer, operation eller sygdom.

Nedsat kulhydrattolerance.

Manifestation af latent diabetes mellitus.

Øget behov for insulin eller orale hypoglykæmiske lægemidler ved diabetes.

Hirsutisme.

*Oftalmologisk:*

Posterior subkapsulær katarakt.

Øget intraokulært tryk.

Glaukom.

Exophthalmus.

*Metabolisk:*

Negativ nitrogenbalance pga. proteinkatabolisme.

*Kardiovaskulært:*

Myokardieruptur efter nylig myokardieinfarkt (se 4.4 Særlige advarsler og forsigtighedsregler vedrørende brugen).

*Andre:*

Hypersensitivitet.

Thromboembolisme.

Vægtøgning.

Øget appetit.

Kvalme.

Utilpashed.

Hikke.

4.9 Overdosering

Rapporter om akut toksicitet og/eller død efter overdosering af glucokortikoi­der er sjældne. Der er ingen specifikke rekommandationer for behandling af overdosering af Decadron.

4.10 Udlevering

B

**5. FARMAKOLOGISKE OPLYSNINGER**

5.0 Terapeutisk klassifikation

H 02 AB 02

5.1 Farmakodynamiske egenskaber

Kortikosteroider har mange og vidtspredte effekter. Disse effekter inkluderer ændringer i kulhydrat-, protein- og lipidmetabolisme; vedligeholdelse af væske og elektrolytbalance; bevarelse af en normal funktion af det kardiovaskulære system, immunsystemer, nyrerne, musklerne, det endokrinologiske system og nervesystemet.

Den farmakologiske og fysiologiske effekt af glukokortikoider synes at formidles af sammen receptor. Derfor har de forskellige syntetiske glukokorti­koider bivirkninger med hensyn til fysiologiske processer der er parallelle til deres terapeutiske effekt. Kortikosteroider er grupperet i forhold til deres relative styrke med henblik på natriumretention, effekt på kulhydratmetabolis­me og antiinflammatorisk effekt.

Dexamethason er en syntetisk adrenokortikal steroid der besidder basisk glukokortikoid aktivitet og virkning. Det er blandt de mest aktive i sin klasse og ca. 25-30 gange så potent som hydrokortison. Ved ækvipotente antiin­flammatoriske doser mangler dexamethason næsten fuldstændig hydrokortison og tæt relaterede hydrokortisonderivaters natriumbesparende egenskab.

5.2 Farmakokinetiske egenskaber

*Distribution:*

En in vitro-undersøgelse viste en gennemsnitsbinding på 77,4% af dexametha­son til plasmaproteiner. Dexamethason bandt sig imidlertid ikke i signifikant grad til kortikosteroidbindende globulin (transcortin) i humant plasma og konkurrerede ikke med cortisol i proteinbinding. Den manglende konkurrence indikerede at de syntetiske steroiders biologiske effekt ikke skyldes frigørelsen af bundet cortisol. Dexamethasons binding til 4% human plasma albuminop­løsninger var 83,7% og bindingen til albumin blev ikke påvirket i dexametha­sonkoncentrationer varierende fra 0,03 til 5,45 mcg/ml. Bindingen til andre non-albumine human plasma proteiner var 6,5% i dexamethasonkoncentratio­ner varierende fra 0,2 til 1,8 mcg/ml. Gelfiltrationsundersøgelser viste, at den konstante affinitet af albumin-dexamethasonkomplekset er underordnet transcortin-cortisolkomplekset.

*Metabolisme:*

Dexamethason største metabolisme bane synes at involvere dannelsen af ukonjugerede polmetabolitter. In vitro-undersøgelser der anvender humane levermikrosomer indikerer at cytokrom P450 3A4 er involveret i dexametha­sonmetabolismen for at danne store polære metabolitter. Disse ukonjugerede polmetabolitter, der inkluderer 6-beta-hydroxydexamethason og konjugeret dexamethason, blev observeret i urinprøver fra patienter der modtog dexamethason

*Udskillelse:*

Dexamethason og dets metabolitter udskilles hovedsageligt gennem nyrerne.

*Kliniske undersøgelser*

Dexamethason har vist sig at være effektivt i forskellige regimer til behandling af kvalme og opkastning efter cisplatin- og non-cisplatinkemoterapi. I nogle kliniske undersøgelser blev der givet dexamethason 8 mg oralt dagen før kemoterapien. Sædvanligvis blev der givet dexamethason 8-20 mg som intravenøs infusion over 5-15 minutter umiddelbart inden kemoterapi. Derefter modtog patienterne sædvanligvis dexamethason oralt, enten som 4 mg hver 4. til 6. time eller som 8 mg hver 8. time, gradvist nedtrappet enten i styrke eller administreringsfrekvens over flere dage. Alternativt blev der givet dexamtha­son intravenøst i stedet for oralt, efter forskellige skemaer.

Dexamethason og et andet potent antiemetisk præparat, ondansetron, var lige effektive med henblik at kurere eller reducere emesisepisoder hos patienter, der modtog non-cisplatin kemoterapi. Selvom dexamethason var lidt mindre effektivt end ondansetron i behandlingen af akutte emesisepisoder, var det mere effektivt i ameliorationen af forsinket emesis. Dexamthason og ondansetron var lige effektivt til behandlingen af akut kvalme, men dexame­thason var mere effektivt som profylakse eller til forsinket kvalme.

Det er påvist, at dexamethason administreret samtidig med ondansetron forstærker effekten af antiemetisk profylakse ved cisplatin- eller non-cisplatin emetogen kemoterapi. Den anvendte dexamthasondosis i kombinationsbehand­lingen svarede til dosis når dexamethason administreres alene. Kombinationen af dexamethason og ondansetron var betydeligt mere effektiv en ondansetron alene til forebyggelsen af cisplatininduceret emesis og kombinationen var associeret med færre bivirkninger end ondansetron alene.

Blandinger med 8 mg ondansetron og 20 mg dexamethasonphosphat, i 50 ml af 5% dextroseinfusionsvæske opbevaret i 50 ml polyvinylchloridinfusionspo­ser har vist sig at være fysisk og kemisk forligelige i op til 2 dage ved almindelig temperatur eller i op til 7 dage ved 2-8° C. Dertil kommer at denne blanding har vist sig at være forligelig med Cortinu-Flo administrerings­sæt.

Det har i kliniske afprøvninger vist sig at samtidig administrering af dexamethason og metoclopramide, i forskellige behandlingsregimer, forstærker effekten af antiemetisk profylakse under emetogen kemoterapi.

5.3 Prækliniske oplysninger

Oral LD_50_ for dexamethason hos hunmus var 6,5 g/kg.

**6. FARMACEUTISKE OPLYSNINGER**

6.1 Indholdsstoffer

Lactosemonohydrat 97,5 mg, calciumhydrogenphosphat, majsstivelse, magnesiumstearat, renset vand.

6.2 Uforligeligheder

Ingen.

6.3 Opbevaringstid

3 år.

6.4 Særlige opbevaringsforhold

Ingen.

6.5 Emballage

Tabletglas.

6.6 Instruktioner vedrørende håndtering

Ingen.

7. Registreringsindehaver

Merck Sharp & Dohme

Waarderweg 39

2003 P.C. Haarlem

Holland

Repræsentant

Merck Sharp & Dohme

Smedeland 8

2600 Glostrup

8. Markedsføringstilladelse nr.

02715

9. Dato for første markedsføringstilladelse

13 oktober 1958

10. Revision af produktresumé

14. september 1999

1. Bezeichnung des Fertigarzneimittels

Cortirel

Wirkstoff: Corticorelin (human)-triflutat

2. Verschreibungsstatus/

Apothekenpflicht

Verschreibungspflichtig

3. Zusammensetzung des Arzneimittels

3.1 Stoff- oder Indikationsgruppe

synthetisches Polypeptid (Releasing Hormon)/

Hypophysenfunktionsdiagnostikum

3.2 Arzneilich wirksame Bestandteile

Eine Injektionsflasche Cortirel mit 10,1 mg

Trockensubstanz enthält 110 bis 121 µg

Corticorelin (human)-triflutat, entsprechend

100 µg Corticorelin (human).

Aminosäuresequenz von Corticorelin (human):

H2N-Ser-Glu-Glu-Pro-Pro-Ile-Ser-Leu-Asp-

Leu-Thr-Phe-His-Leu-Leu-Arg-Glu-Val-Leu-

Glu-Met-Ala-Arg-Ala-Glu-Gln-Leu-Ala-Gln-

Gln-Ala-His-Ser-Asn-Arg-Lys-Leu-Met-Glu-

Ile-Ile-NH2

3.3 Sonstige Bestandteile

Eine Injektionsflasche mit 10,1 mg Trockensubstanz

enthält Mannitol.

Eine Injektionsflasche mit 1 ml Lösungsmittel

(pH 2,3) enthält Wasser für Injektionszwecke,

Natriumchlorid 0,009 g sowie Salzsäure

zur pH-Einstellung.

4. Anwendungsgebiete

Überprüfung der corticotropen Partialfunktion

des Hypophysenvorderlappens in allen

Fällen, in denen eine organische Schädigung

dieser Funktion vermutet werden

kann, also u. a. bei Patienten mit Hypophysentumoren,

Craniopharyngeom, bei Patienten

mit Verdacht auf Hypophyseninsuffizienz,

Panhypopituitarismus, Syndrom der leeren

Sella, sowie bei Patienten mit traumatischen

und postoperativen Schädigungen der Hypophysenregion

und Patienten, bei denen

eine Bestrahlung der Hypophysenregion erfolgt

ist.

Auch funktionelle Störungen wie z. B.

nach längerer Glucocorticoid-Medikation

(systemisch oder lokal) werden mit Cortirel

überprüfbar.

5. Gegenanzeigen

Bekannte Allergie gegen Corticorelin (human).

Wegen unzureichender Erfahrung bei der

Anwendung im Kindesalter sind Kinder von

der Behandlung zunächst ausgenommen.

Eine Dosisempfehlung fehlt noch für diese

Gruppe von Patienten.

Schwangerschaft und Stillzeit

Mit der Anwendung in Schwangerschaft und

Stillzeit bestehen keine Erfahrungen.

6. Nebenwirkungen

Gelegentlich können ein leichtes Wärmegefühl

im Kopf-, Hals- und Oberkörperbereich,

sowie leichte Geruchs- und Geschmackssensationen

auftreten, die rasch abklingen.

Die Möglichkeit des Auftretens einer allergischen

Reaktion kann nicht ausgeschlossen

werden.

7. Wechselwirkungen mit

anderen Mitteln

Die gleichzeitige Anwendung von Arzneimitteln,

die die Wirkung von Cortirel hemmen

können, wie Corticosteroide, Antihistaminika,

Antiserotoninergika oder Oxitocin und/

oder die gleichzeitige Anwendung von Arzneimitteln,

die die Wirkung von Cortirel

verstärken können, wie Vasopressin und

seine Analogen, ist zu vermeiden.

8. Warnhinweise

keine

9. Wichtigste Inkompatibilitäten

Cortirel sollte nicht gemeinsam mit anderen

Parenteralia (z. B. in Mischspritzen oder

Infusionslösungen) verabreicht werden.

10. Dosierung mit Einzel- und Tagesgaben

Der Inhalt einer Injektionsflasche Cortirel

[110 – 121 µg Corticorelin (human)-triflutat]

gelöst in 1 ml des beiliegenden Lösungsmittels

gilt als Dosierung bei normalgewichtigen

erwachsenen Patienten. Bei stark übergewichtigen

Patienten kann nach der Gewichts-

Dosis-Relation vorgegangen werden,

d. h. es werden 2 µg/kg Körpergewicht injiziert.

Nach Anbruch sind die Reste zu verwerfen.

11. Art und Dauer der Anwendung

Zur Bestimmung der basalen Corticotropin-

(ACTH) und Cortisolspiegel in Serum oder

Plasma wird ca. 2 ml Venenblut abgenommen.

(Beachten Sie unbedingt die Hinweise

des Labors, welches die Bestimmung

durchführt bezüglich der Probengewinnung

und Lagerung: in der Regel EDTA-Plasma,

gekühlt verarbeitet, über Trockeneis tiefgefroren

transportiert).

Der Inhalt einer Injektionsflasche Cortirel

wird in 1 ml des beiliegenden Lösungsmittels

gelöst und als Bolusinjektion innerhalb

von ca. 30 Sekunden intravenös verabreicht.

Die Abnahme einer weiteren Probe von

Venenblut sollte 30 Minuten nach der i.v. Applikation

erfolgen.

Zur besseren Beurteilung des Anstieges der

ACTH- und Cortisolspiegel können 15, 45,

60 und 90 Minuten nach i.v. Applikation zusätzliche

Blutproben entnommen werden.

Die Anwendung ist als einmaliger Test vorgesehen.

Eine Wiederholung des Tests sollte

nur bei klinisch begründeten Fällen auf

besondere Anordnung des Arztes erfolgen.

12. Notfallmaßnahmen, Symptome und

Gegenmittel

Sollte es zur allergischen Reaktion kommen,

sind sofort Gegenmaßnahmen zu ergreifen:

0,5 ml Epinephrin (Adrenalin) 1: 1000 s.c.,

gegebenenfalls nach 5 Minuten Wiederholung

der Gabe; in schweren Fällen (allergischer

Schock) ist die i.v. Gabe von

0,25 – 1 ml Epinephrin 1: 10.000, d. h. eine

1: 10 Verdünnung der handelsüblichen Epinephrinlösung

(1 ml Epinephrinlösung +

9 ml physiologische Kochsalzlösung) angezeigt,

die gegebenenfalls wiederholt werden

kann.

Eine als unangenehm empfundene Blutdrucksenkung

kann durch Hochlagern der

Beine und i.v. Zufuhr von Flüssigkeit kompensiert

werden.

Symptome der Intoxikation

Bei höheren Dosierungen kann es zu episodischem

Blutdruckabfall mit beschleunigter

Herztätigkeit und erhöhter Prolaktinausschüttung

kommen.

13. Pharmakologische und toxikologische

Eigenschaften, Pharmakokinetik und

Bioverfügbarkeit, soweit diese Angaben

für die therapeutische Verwendung

erforderlich sind

13.1 Pharmakologische Eigenschaften

Corticorelin (human), das auch natürlich im

menschlichen Organismus vorkommt, bewirkt

physiologisch die Sekretion von adrenocorticotropem

Hormon (ACTH) aus der

Hypophyse gefolgt von der Ausschüttung

von Cortisol aus der Nebennierenrinde.

13.2 Toxikologische Eigenschaften

Bei Prüfung von Corticorelin (human)-triflutat

auf akute Toxizität wurden nach i.v. Injektion

der ca. 100fachen klinischen Dosis (143 µg/

kg) sowohl bei Ratten (5 männliche/5 weibliche)

als auch bei Kaninchen (3 männliche)

weder lokale noch systemische Wirkungen

beobachtet. Auch bei der wiederholten Anwendung

der 100fachen klinischen Dosis i.v.

über 5 Tage traten bei Mäusen (5 männliche/

5 weibliche) weder lokale noch systemische

Wirkungen und keine makroskopisch-pathologischen

Befunde auf.

13.3 Pharmakokinetik

Nach intravenöser Applikation von

110 – 121 µg Corticorelin (human)-triflutat

steigen beim Menschen die Konzentrationen

von Corticorelin (human) im Plasma innerhalb

von 5 Minuten auf Maximalwerte an

und fallen anschließend wieder ab. Die Halbwertzeit

für eine 110 – 121 µg Dosis Corticorelin

(human)-triflutat beträgt 9 Minuten.

14. Sonstige Hinweise

keine

15. Dauer der Haltbarkeit

Die Trockensubstanz ist bei Lagerung von

2C–8C 3 Jahre haltbar.

Cortirel soll nach Ablauf des auf der Pakkung

angegebenen Verfalldatums nicht

mehr angewendet werden. Die gebrauchsfertige

Lösung sollte sofort verwendet werden.

16. Besondere Lagerund

Aufbewahrungshinweise

Bei 2C–8C aufbewahren!

August 2004

Fachinformation

Cortirel

1 8511-w838 -- Cortirel -- a

17. Darreichungsformen, Packungsgrößen

Einzelpackung N 1

1 Injektionsflasche mit 110 – 121 µg Corticorelin

(human)-triflutat und Mannitol als Trokkensubstanz

und 1 Injektionsflasche mit

1 ml 0,9 % Natriumchlorid-Lösung (pH 2,3)

Großpackung N 2

5 Injektionsflaschen mit je 110 – 121 µg Corticorelin

(human)-triflutat und Mannitol als

Trockensubstanz und 5 Injektionsflaschen

mit je 1 ml 0,9 % Natriumchlorid-Lösung

(pH 2,3)

18. Stand der Information

August 2004

19. Name oder Firma und Anschrift

des pharmazeutischen

Unternehmers

Curatis Pharma GmbH

Karl-Wiechert-Allee 76

30625 Hannover

Tel.: 0511/5304511

Fax: 0511/5304510

E-mail: officecuratis-pharma.de

Vertrieb:

Meduna Arzneimittel GmbH

Ernst-Grote-Str. 23

D-30916 Isernhagen

Tel.: 0511/6151372

Fax: 0511/6151375

Zentrale Anforderung an:

Rote Liste Service GmbH

FachInfo-Service

Postfach 11 01 71

10831 Berlin

Fachinformation

Cortirel

2 8511-w838 -- Cortirel – a
